# Supplementary material for: Preclinical Detection of Early Glomerular Injury in Children with Kidney Diseases—Independently of Usual Markers of Kidney Impairment and Inflammation
Source: Int J Mol Sci. 2024 Aug 28;25(17):9320. doi: 10.3390/ijms25179320 (PMC11395411; doi:10.3390/ijms25179320)

SUPPLEMENTARY ITEMS

SUPPLEMENTARY METHODS

SDS transformation of blood pressure and BMI

In all children height, body mass index and blood pressure are dynamic parameters and change physiologically with age. Therefore, unlike as in adults, absolute parameters cannot be compared directly between children of different ages and sexes. To correct for these physiological differences, we calculated Standard Deviation Scores (SDS; also known as z-scores) as follows:

(y-m)/SD

where y is the value for an individual patient, m is the mean of the control population and SD is the standard deviation of the control population.

Supplementary Table S1: Specification of ELISA-Test kits applied

The assays were performed according the suppliers instructions with the following modifications: all assays from Cloud-Clone and CUSBIO were performed with double incubation time to increase sensitivity. Samples were pre-incubated with 0.5% Tween 20 before measured of gelsolin to reach dilutional linearity. For the quantification of PICP, TGF-β1, and CRP in urine either incubation times were prolonged or concentrated specimens were applied. For that samples have been concentrated in Amicon® Ultra -15 Centrifugal filters (3K, UFC900324) by ~10 to ~60 fold depending on the starting volume available.

SUPPLEMENTARY RESULTS

Supplementary Figure S1: Comparison of the concentrations of BM candidates in serum and urine from patients and controls

Entire results are given here.

Significance values are given as bars: $p < 0.05$ (bright blue); $p < 0.01$ (dark blue); all other not significant.

First series: biomarker concentrations in serum (S)

Second series: biomarker concentrations in urine (U)

Abbreviations of biomarker proteins: cf. main text

Supplementary Table S2: Correlation of each BM in urine with each other's

Analyses were performed by SPSS (v.27). Lower left halve: correlation according Pearson; upper right halve: correlation according Spearman. Each cell shows the correlation coefficient (upper figure) and a p-value (lower figure).

* and ** indicate significant and highly significant correlations proposed by SPSS. Notwithstanding that, we set a correlation coefficient >0.500 moderately significant and >0.600 highly significant and mark these values by blue (Perason) and green (Spearman) filling.

ROC analysis, comparison of BM values from patient´s groups with healthy controls (Tables S3 to S8)

Table S3: Patients with obesity, hypertension, metabolic syndrome, and T2DM (group A)

Table S4: Patients with T1DM (group B)

Table S5: Patients with renal agenesis, hypoplasia, multicystic renal dysplasia (group C)

Table S6: Patients with anomalies of the urinary tract, duplex kidneys (group D)

Table S7: Patients with post-infectious GN, IgAN, IgAV (group G)

Table S8: Entire nephropathy patients (group A to G)

Correlation analyses of BMs with individual parameters

Sub-group of healthy controls (Table S9 to S11)

Table S9: Correlation of serum and urinary BM candidates with BMI z-score

Table S10: Correlation of serum and urinary BM candidates with age

Graphical visualization of correlations considered significant
Serum angiotensinogen and complement component C1q with age

Table S11: Correlation of serum and urinary BM candidates with blood pressure (BP z-score)

Graphical visualization of the correlation considered significant
Serum adiponectin with diastolic blood pressure

Patients with obesity, hypertension, metabolic syndrome, and T2DM (group A, Tables S12 to S15)

Table S12: Correlation of serum and urinary BM candidates with UACR and blood glucose

Graphical visualization of correlations considered as significant:
Urinary angiotensinogen (AGT), gelsolin (GS), vitronectin (VTN), and complement component C9 (C9) with blood glucose concentration

Table S13: Correlation of serum and urinary BM candidates with HbA1c and BMI z-score

Table S14: Correlation of serum and urinary BM candidates with serum and urinary CRP

Graphical visualization of correlations considered as significant:
Urinary gelsolin (GS) with serum CRP, and urinary CRP with serum CRP

Table S15: Correlation of serum and urinary BM candidates with blood pressure(BP z-score)

Graphical visualization of correlations considered as significant:
Urinary adiponectin (UADP) and serum complement factor I (SCFI) with systolic blood pressure
Serum PICP, TGFβ, and urinary complement component 9 (UC9) with diastolic blood pressure

Patients with T1DM (group B)

Table S16: Correlation of serum and urinary BM candidates with UACR and blood glucose

Graphical visualization of correlations considered as significant:
Serum CRP, urinary (U) alpha1-acid glycoprotein (a1AGP), and PICP with UACR, as well as serum (S) a1AGP and leucine rich glycoprotein 1 (LRGP1) with blood glucose

Table S17: Correlation of serum and urinary BM candidates with HbA1c and BMI z-scores

Table S18: Correlation of serum and urinary BM candidates with blood pressure (BP z-score)

Table S19: Correlation of serum and urinary BM candidates with disease duration

Patients with nephropathies (groups A to G, Tables S20 to S23)

Table S20: Correlation of serum and urinary BM candidates with serum creatinine and UACR

Graphical visualization of correlation considered as significant:
Urinary lumican (ULUM) with UACR

Table S21: Correlation of serum and urinary BM candidates with eGFR and cGFR

Graphical visualization of correlations considered as significant:
Serum complement factor H with eGFR and cGFR

Table S22: Correlation of serum and urinary BM candidates with serum CRP

Graphical visualization of correlation considered as significant:
Serum complement factor H with serum CRP

Table S23: Correlation of serum and urinary BM candidates with blood pressure (BP z-score)

Analysis of sex differences of BM

Table S24: Significance (p-values) of sex differences of urinary BM in various groups

Table S25: Significance (p-values) of sex differences of serum BM in various groups

BM that appear significantly different between males and females are indicated by bold letters and are shown graphically. Empty cells: not enough values for analysis.

Supplementary Figure S1:
Comparison of the concentrations of BM candidates in serum and urine from patients and controls

Entire results are given here (17 BM candidates each in serum and urine).

Significance values are given as bars.
First series: biomarker concentrations in serum (S)
Second series: biomarker concentrations in urine (U)
Abbreviations of biomarker proteins: cf. main text

P < 0.01
P < 0.05
0.05 < *P* <0.10

Serum

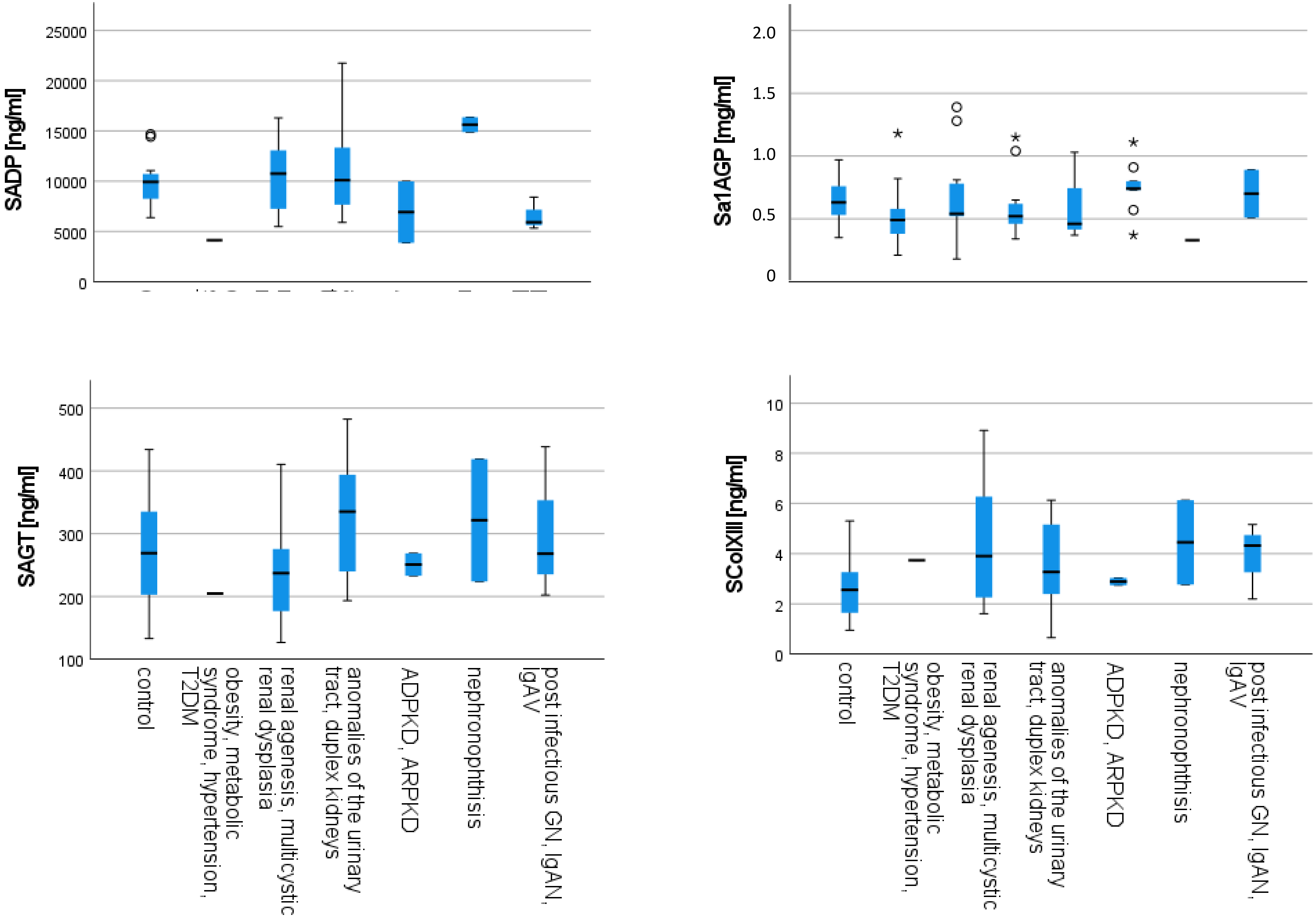
SADP [ng/ml]
25000
20000
15000
10000
5000
0
Sa1AGP [mg/ml]
2.0
1.5
1.0
0.5
0
SAGT [ng/ml]
500
400
300
200
100
SColXIII [ng/ml]
10
8
6
4
2
0
control
obesity, metabolic syndrome, hypertension, T2DM
renal agenesis, multicystic renal dysplasia
anomalies of the urinary tract, duplex kidneys
ADPKD, ARPKD
nephronophthisis
post infectious GN, IgAN, IgAV

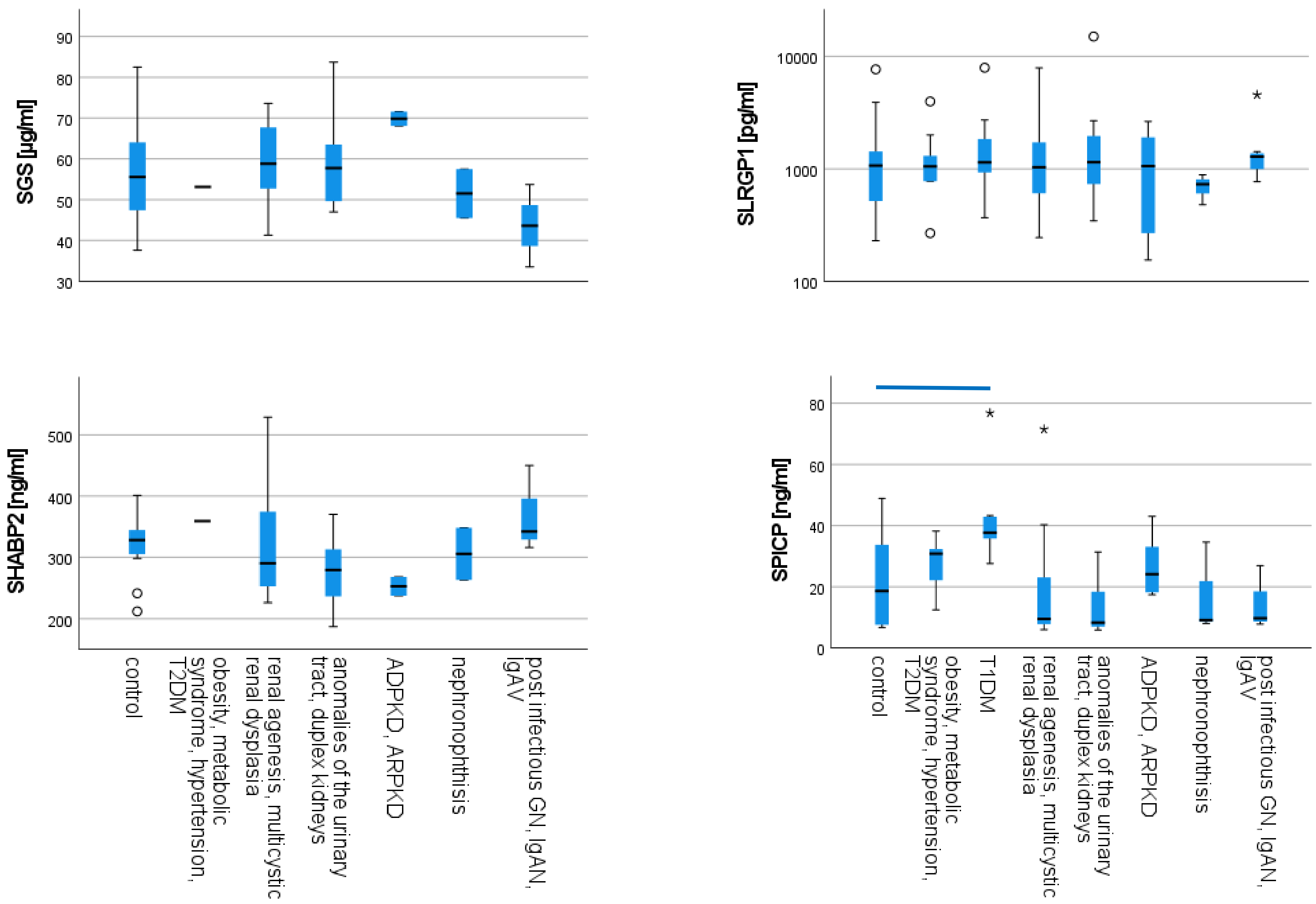
SGS [µg/ml]
90
80
70
60
50
40
30
SLRGP1 [pg/ml]
10000
1000
100
SHABP2 [ng/ml]
500
400
300
200
SPICP [ng/ml]
80
60
40
20
0
control
obesity, metabolic syndrome, hypertension, T2DM
T1DM
renal agenesis, multicystic renal dysplasia
anomalies of the urinary tract, duplex kidneys
ADPKD, ARPKD
nephronophthisis
post infectious GN, IgAN, IgAV

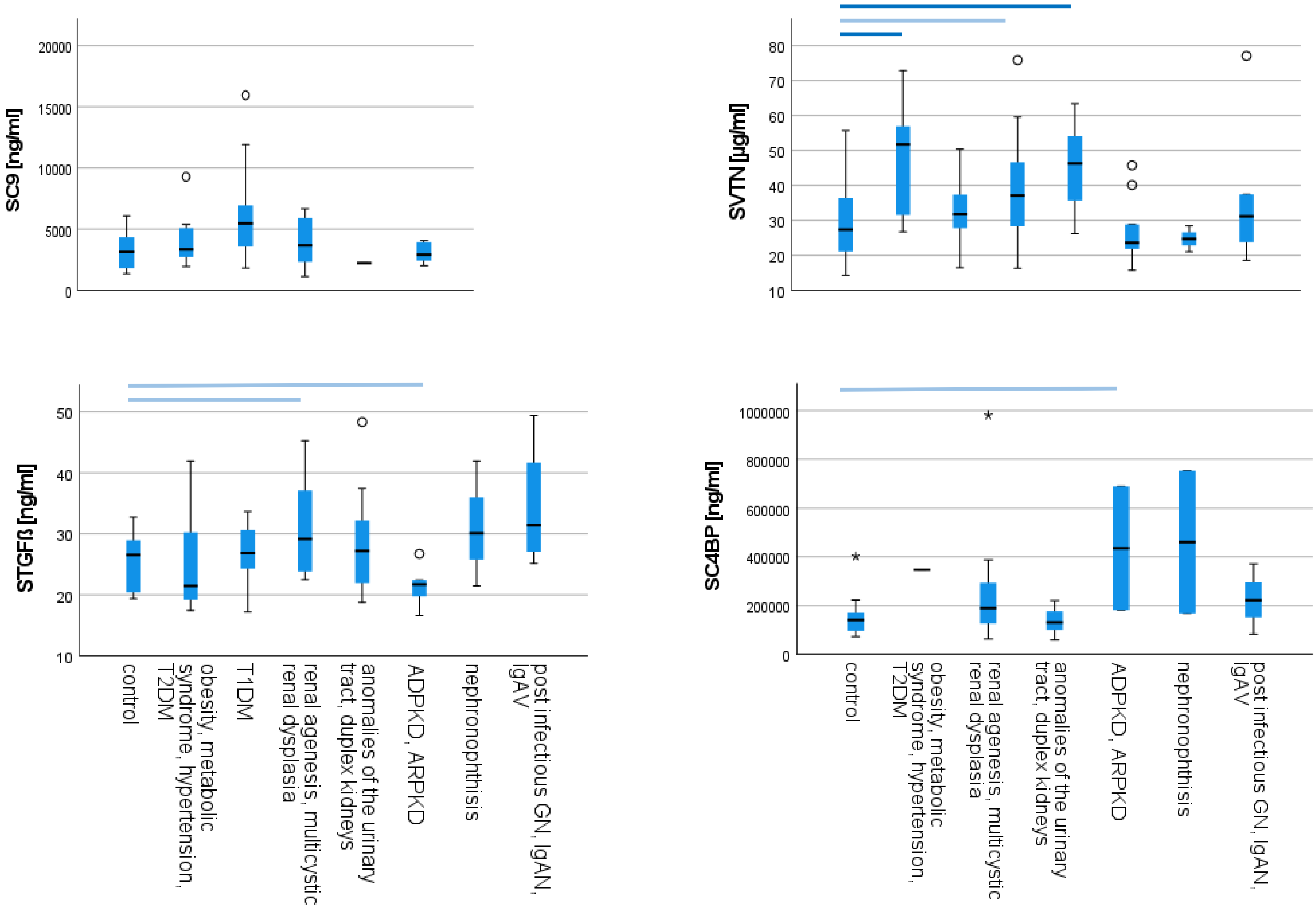
SC9 [ng/ml]
0
5000
10000
15000
20000
SVTN [µg/ml]
10
20
30
40
50
60
70
80
STGFß [ng/ml]
10
20
30
40
50
SC4BP [ng/ml]
0
200000
400000
600000
800000
1000000
control
obesity, metabolic syndrome, hypertension, T2DM
T1DM
renal agenesis, multicystic renal dysplasia
anomalies of the urinary tract, duplex kidneys
ADPKD, ARPKD
nephronophthisis
post infectious GN, IgAN, IgAV

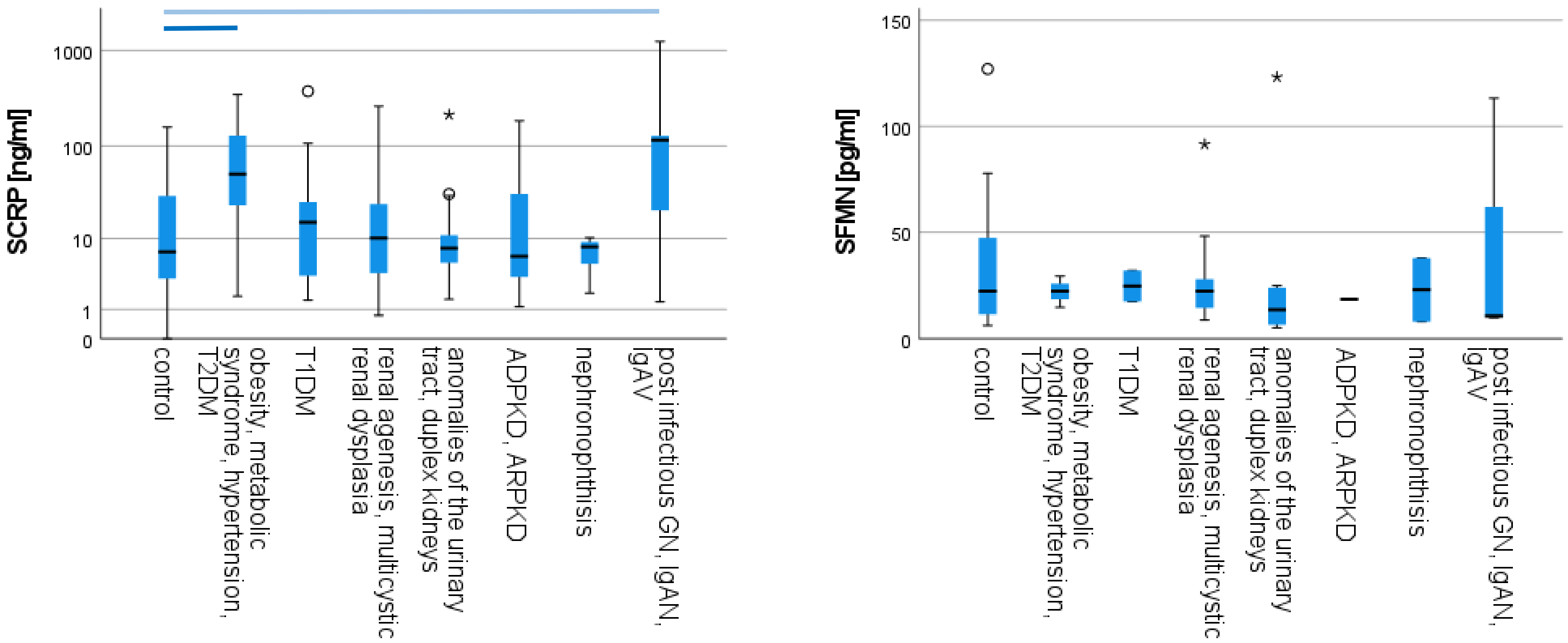

SCRP [ng/ml]
1000
100
10
1
0
control
obesity, metabolic syndrome, hypertension, T2DM
T1DM
renal agenesis, multicystic renal dysplasia
anomalies of the urinary tract, duplex kidneys
ADPKD, ARPKD
nephronophthisis
post infectious GN, IgAN, IgAV
SFMN [pg/ml]
150
100
50
0
control
obesity, metabolic syndrome, hypertension, T2DM
T1DM
renal agenesis, multicystic renal dysplasia
anomalies of the urinary tract, duplex kidneys
ADPKD, ARPKD
nephronophthisis
post infectious GN, IgAN, IgAV

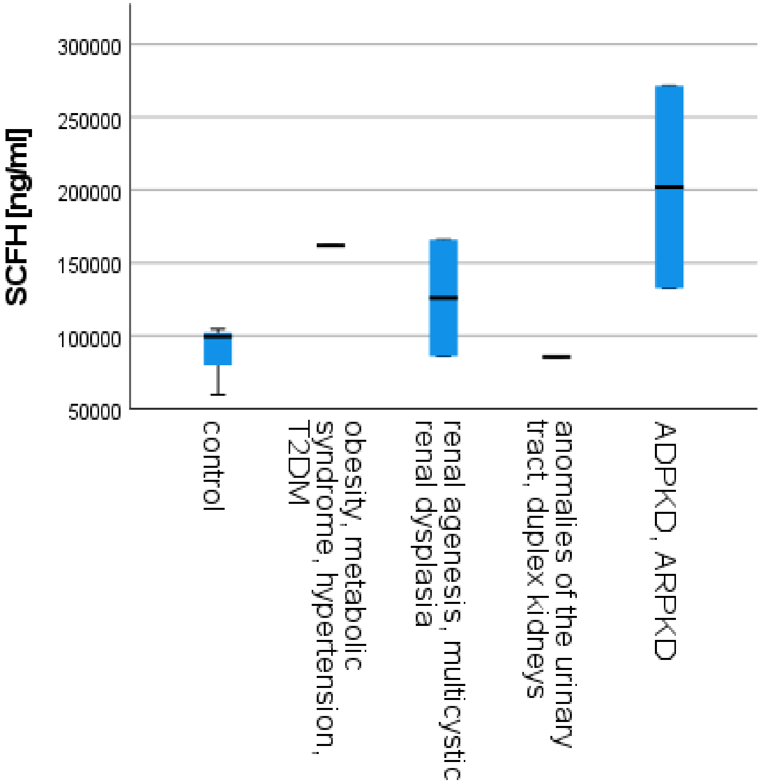
SCFH [ng/ml]
300000
250000
200000
150000
100000
50000
control
obesity, metabolic syndrome, hypertension, T2DM
renal agenesis, multicystic renal dysplasia
anomalies of the urinary tract, duplex kidneys
ADPKD, ARPKD

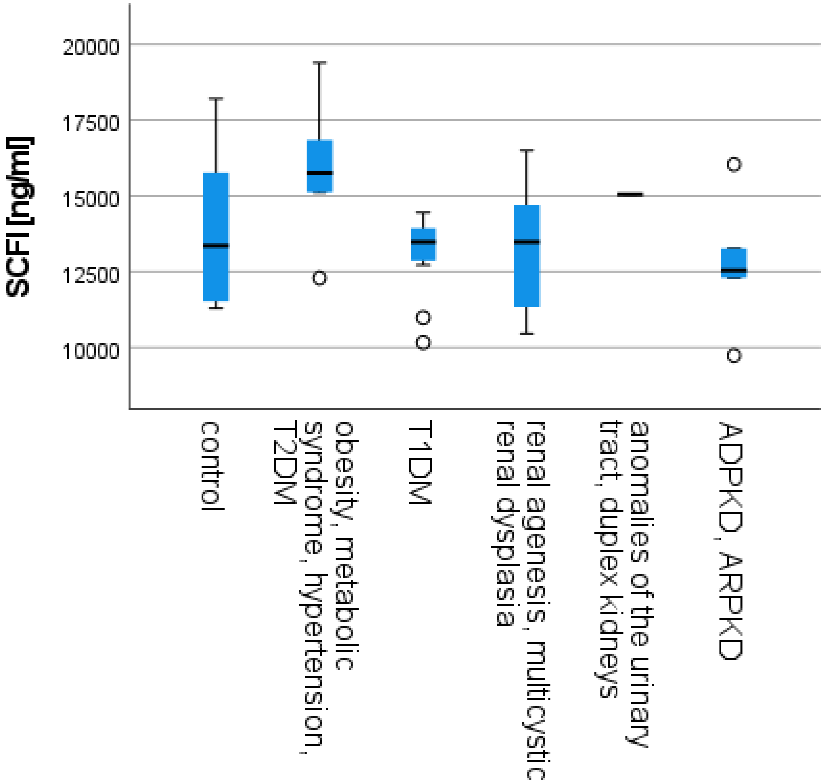
SCFI [ng/ml]
20000
17500
15000
12500
10000
control
obesity, metabolic syndrome, hypertension, T2DM
T1DM
renal agenesis, multicystic renal dysplasia
anomalies of the urinary tract, duplex kidneys
ADPKD, ARPKD

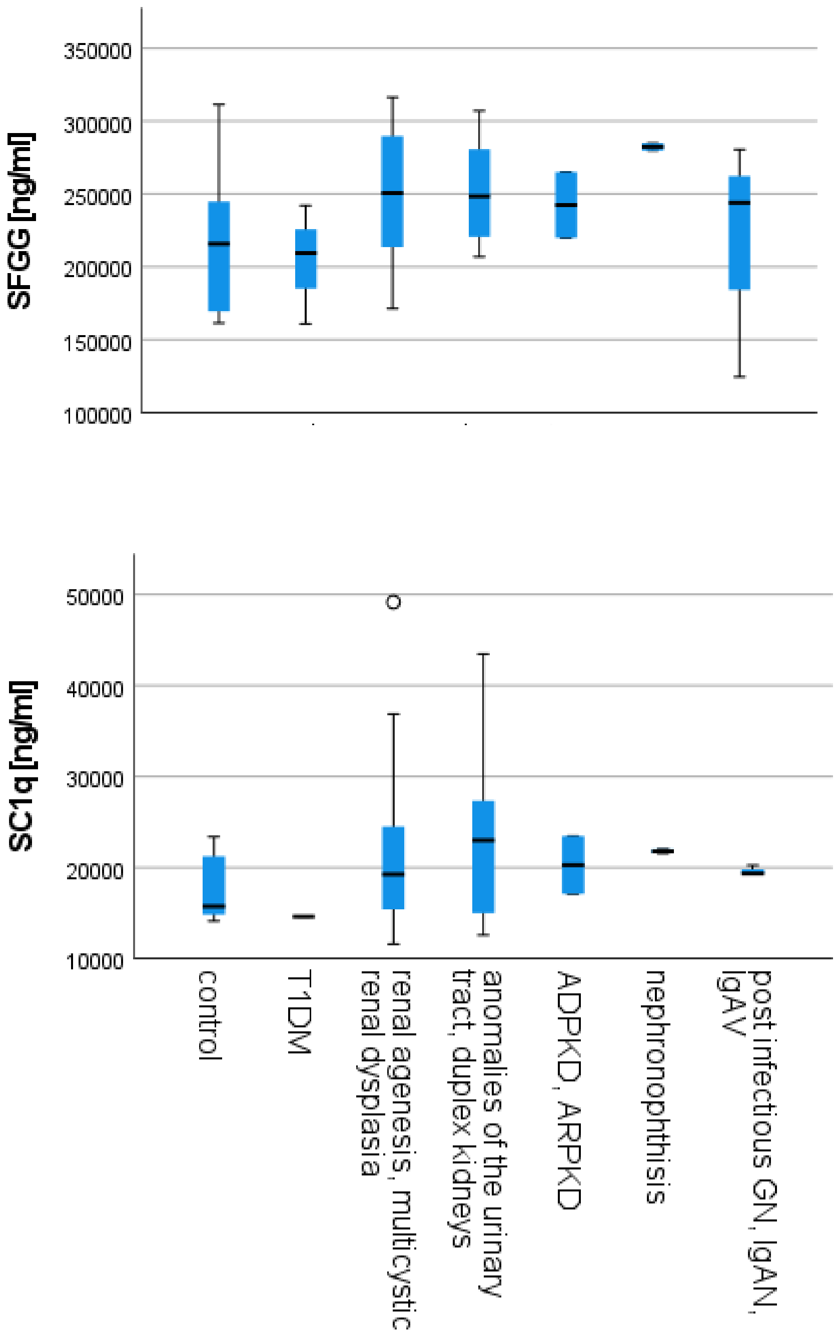
SFGG [ng/ml]
350000
300000
250000
200000
150000
100000
SC1q [ng/ml]
50000
40000
30000
20000
10000
control
T1DM
renal agenesis, multicystic renal dysplasia
anomalies of the urinary tract, duplex kidneys
ADPKD, ARPKD
nephronophthisis
post infectious GN, IgAN, IgAV

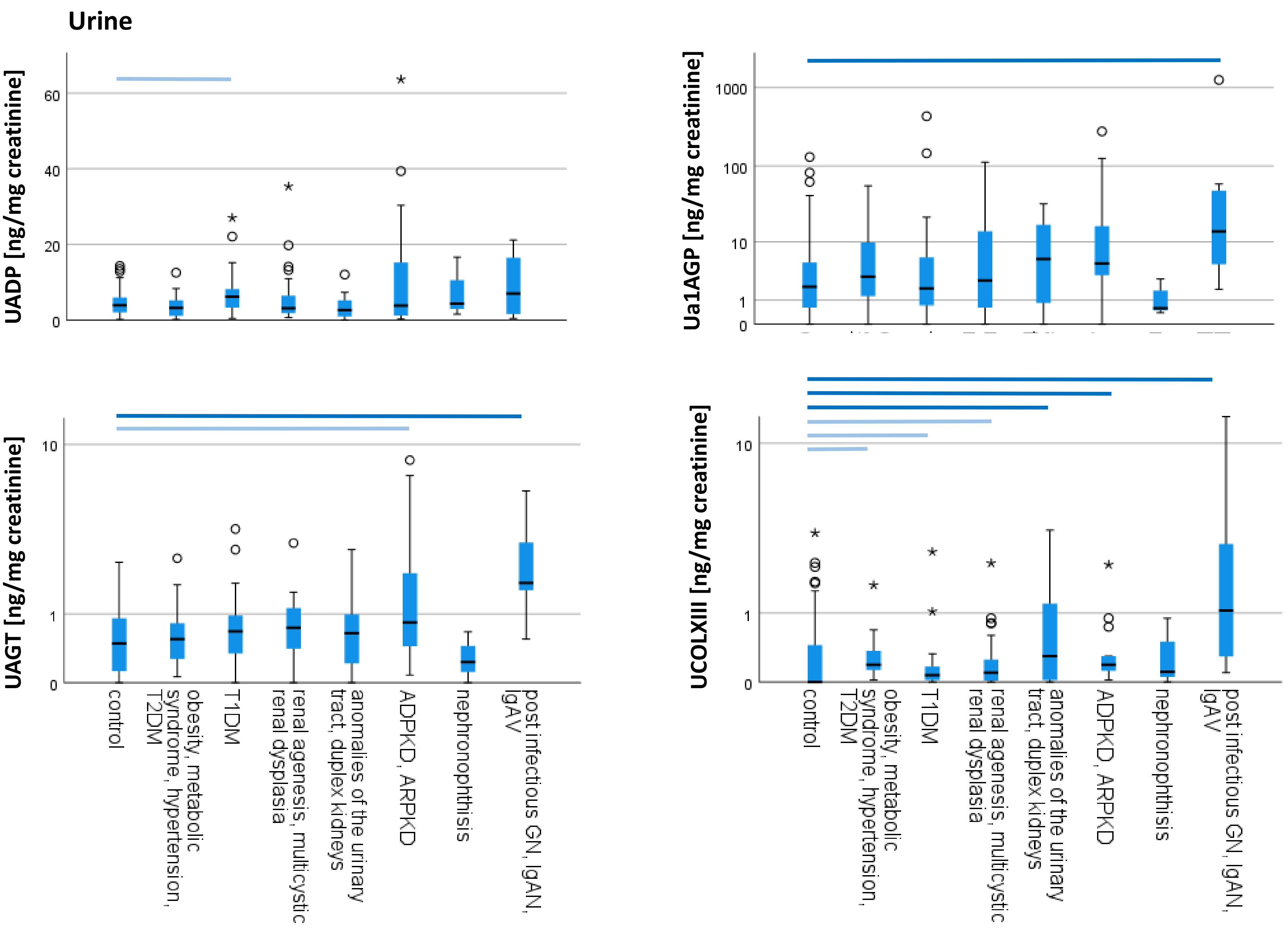
Urine
UADP [ng/mg creatinine]
Ua1AGP [ng/mg creatinine]
UAGT [ng/mg creatinine]
UCOLXIII [ng/mg creatinine]
control
obesity, metabolic syndrome, hypertension, T2DM
T1DM
renal agenesis, multicystic renal dysplasia
anomalies of the urinary tract, duplex kidneys
ADPKD, ARPKD
nephronophthisis
post infectious GN, IgAN, IgAV

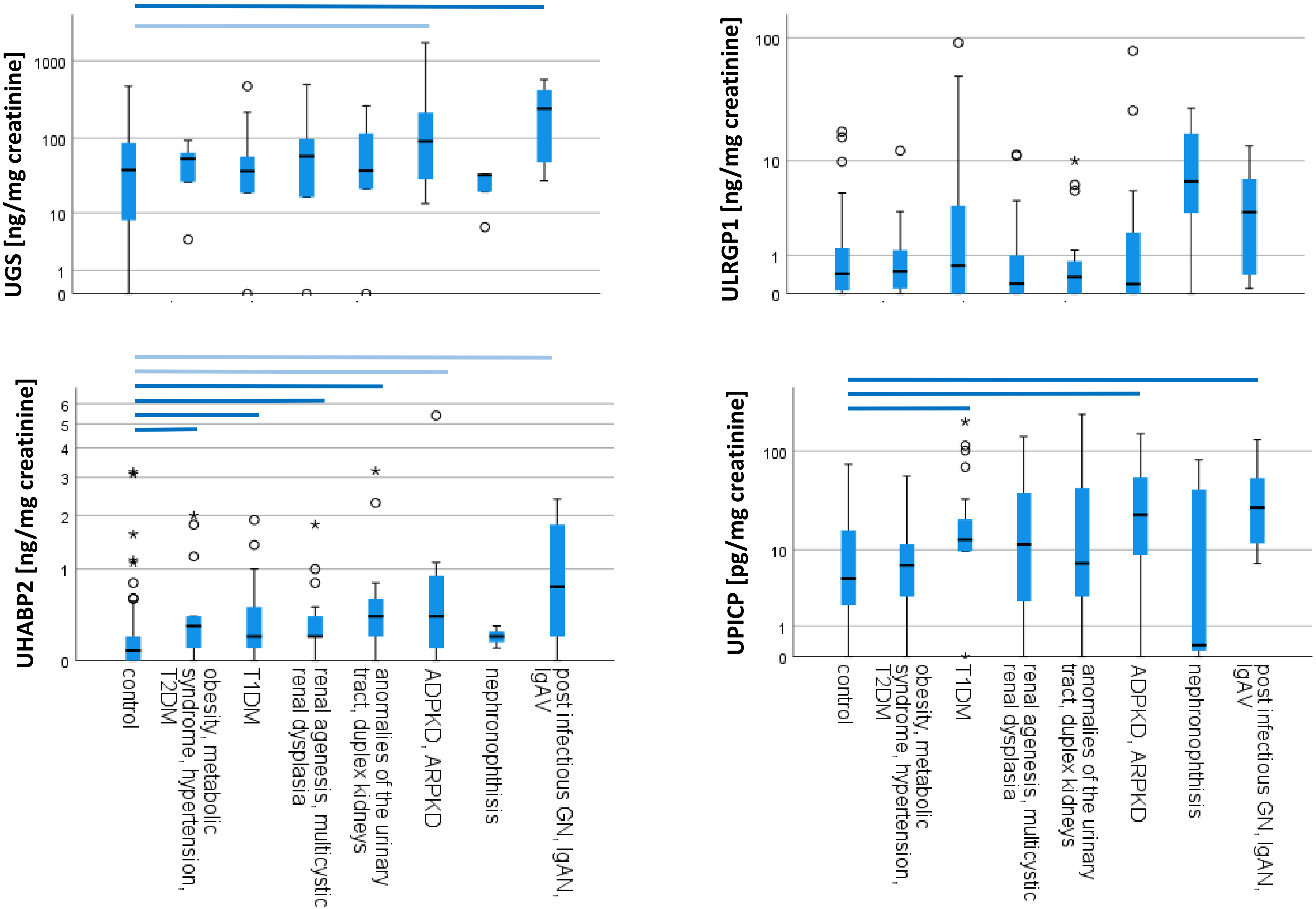

UGS [ng/mg creatinine]
1000
100
10
1
0
ULRGP1 [ng/mg creatinine]
100
10
1
0
UHABP2 [ng/mg creatinine]
6
5
4
3
2
1
0
UPICP [pg/mg creatinine]
100
10
1
0
control
obesity, metabolic syndrome, hypertension, T2DM
T1DM
renal agenesis, multicystic renal dysplasia
anomalies of the urinary tract, duplex kidneys
ADPKD, ARPKD
nephronophthisis
post infectious GN, IgAN, IgAV

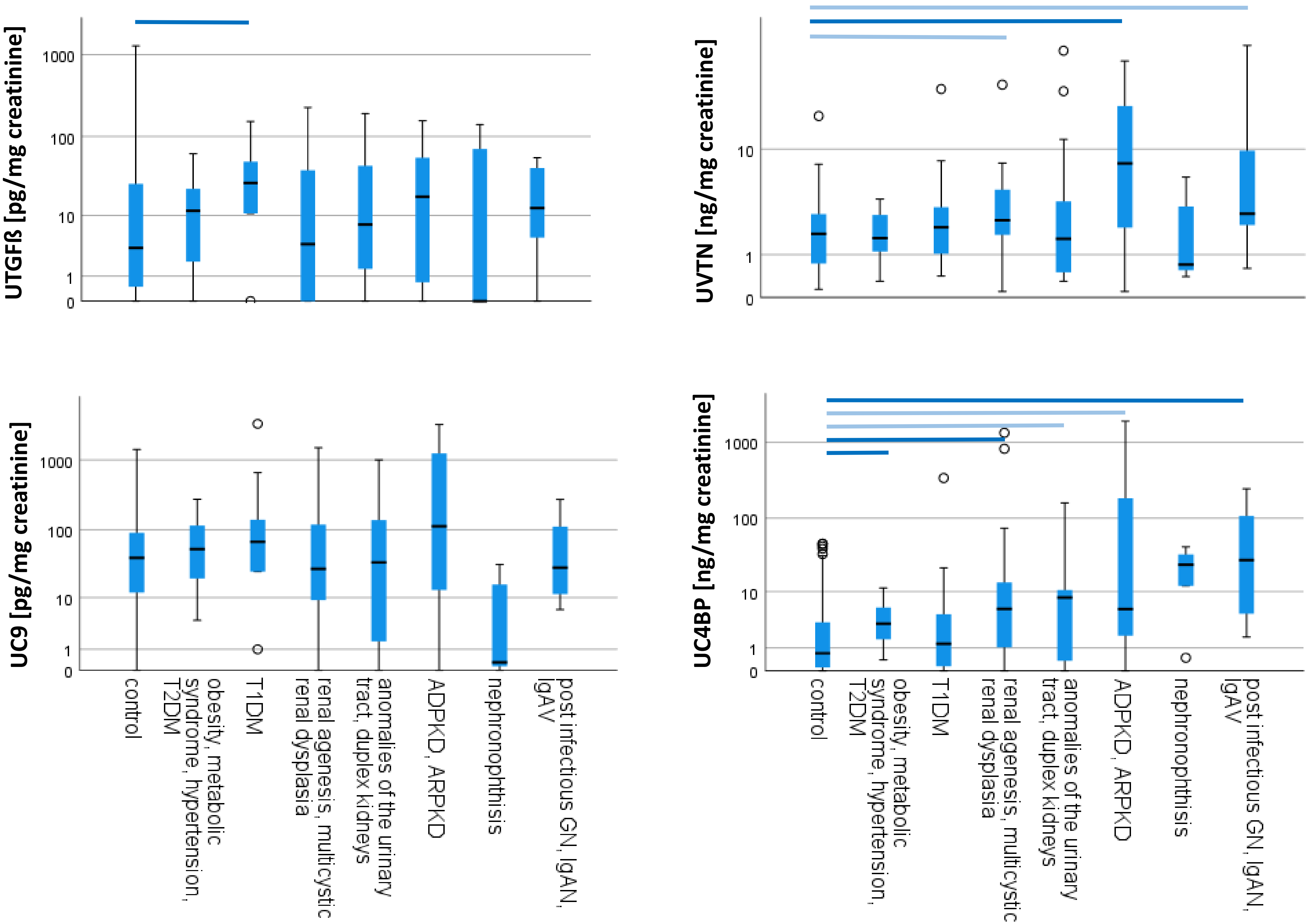
UTGFß [pg/mg creatinine]
1000
100
10
1
0
UVTN [ng/mg creatinine]
10
1
0
UC9 [pg/mg creatinine]
1000
100
10
1
0
UC4BP [ng/mg creatinine]
1000
100
10
1
0
control
obesity, metabolic syndrome, hypertension, T2DM
T1DM
renal agenesis, multicystic renal dysplasia
anomalies of the urinary tract, duplex kidneys
ADPKD, ARPKD
nephronophthisis
post infectious GN, IgAN, IgAV

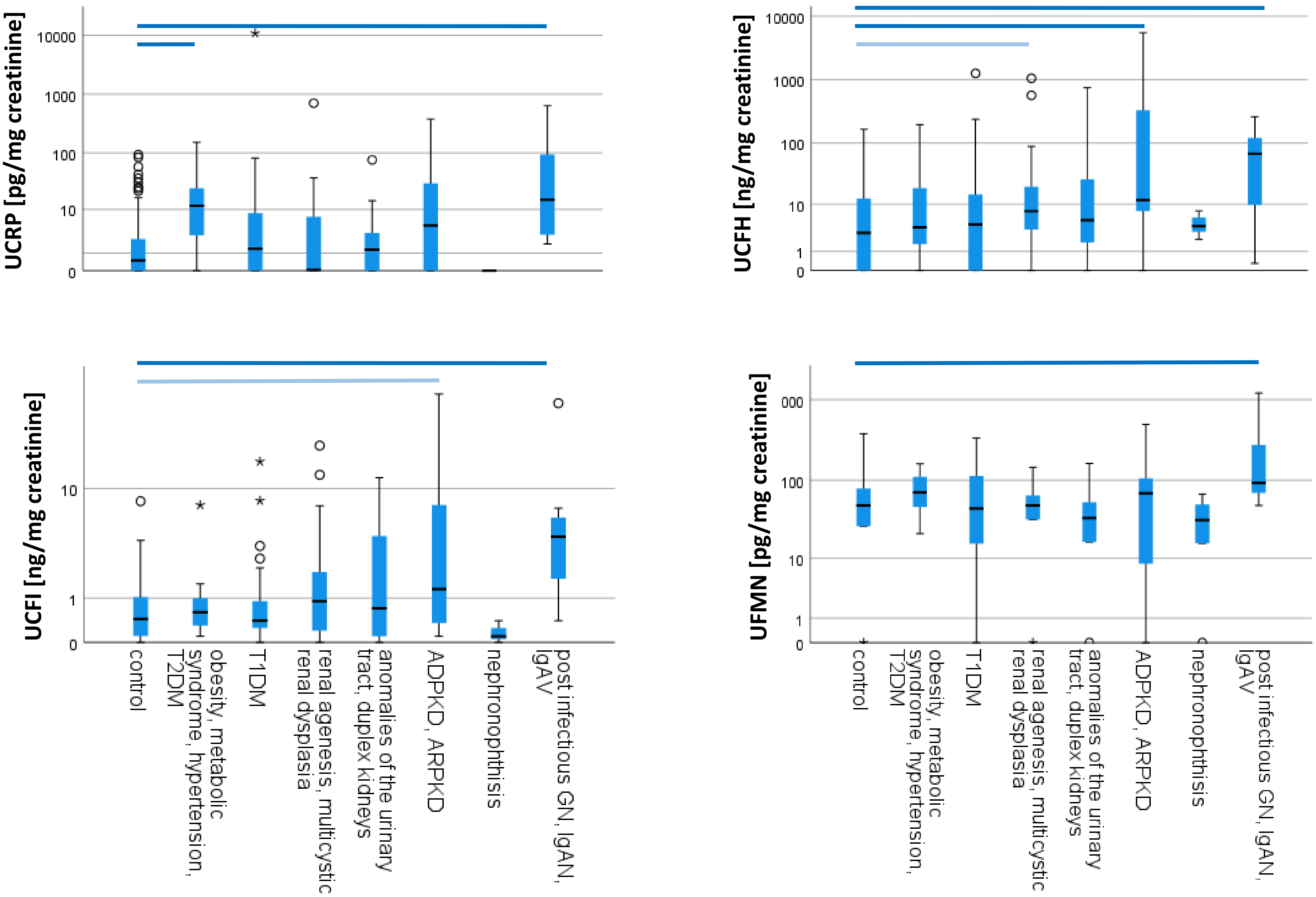
UCRP [pg/mg creatinine]
10000
1000
100
10
0
UCFH [ng/mg creatinine]
10000
1000
100
10
1
0
UCFI [ng/mg creatinine]
10
1
0
UFMN [pg/mg creatinine]
000
100
10
1
0
control
obesity, metabolic syndrome, hypertension, T2DM
T1DM
renal agenesis, multicystic renal dysplasia
anomalies of the urinary tract, duplex kidneys
ADPKD, ARPKD
nephronophthisis
post infectious GN, IgAN, IgAV

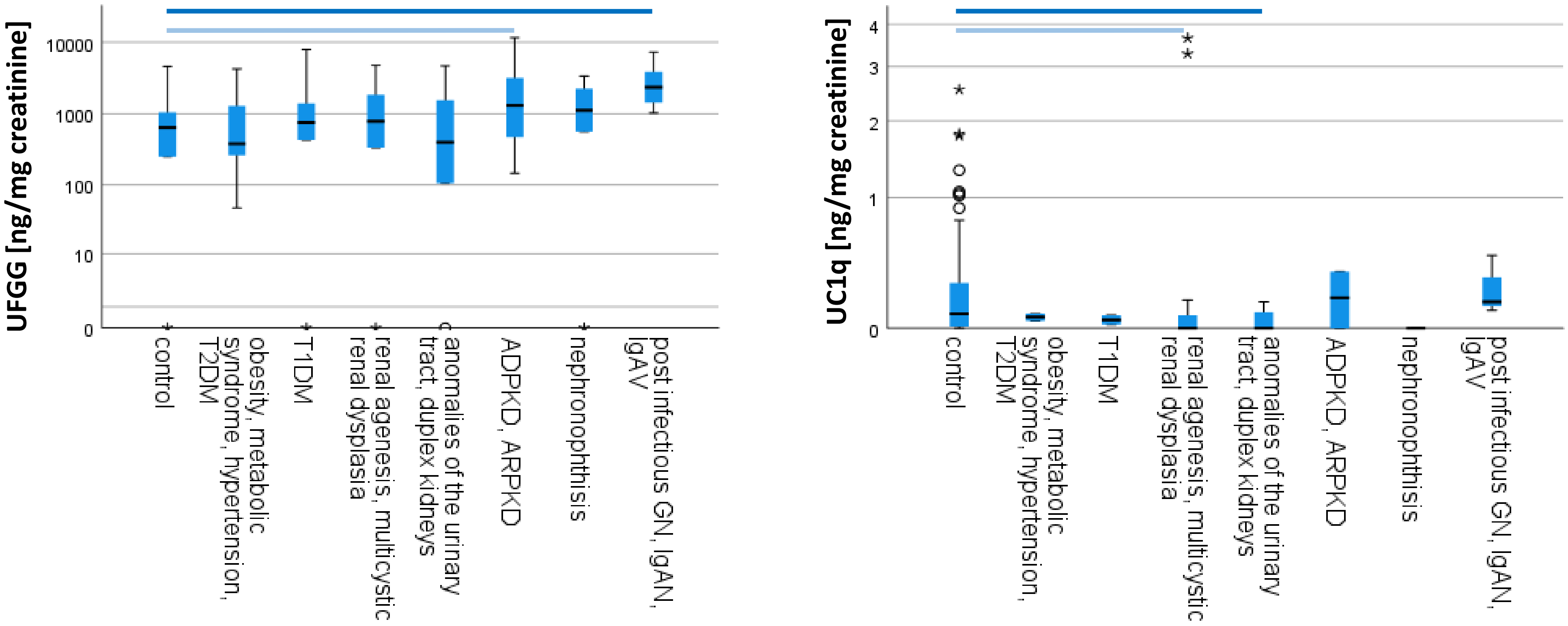
UFGG [ng/mg creatinine]
10000
1000
100
10
0
control
obesity, metabolic syndrome, hypertension, T2DM
T1DM
renal agenesis, multicystic renal dysplasia
anomalies of the urinary tract, duplex kidneys
ADPKD, ARPKD
nephronophthisis
post infectious GN, IgAN, IgAV
UC1q [ng/mg creatinine]
4
3
2
1
0
control
obesity, metabolic syndrome, hypertension, T2DM
T1DM
renal agenesis, multicystic renal dysplasia
anomalies of the urinary tract, duplex kidneys
ADPKD, ARPKD
nephronophthisis
post infectious GN, IgAN, IgAV

Supplementary Table S1: Specification of ELISA-Test kits applied

antigen	supplier	code
angiotensinogen	Abbexa	abx573778
formin 1		abx387383
alpha-1-acid glycoprotein	Cloud-Clone	HEA816Hu
coagulation factor XIIIB		CEB613Hu
complement C1q		SEA747Hu
complement factor H		SEA635Hu
complement factor I		SEB978Hu
collagen type XIII		SEC138Hu
c-reactive protein		SEA821Hu
fibronectin		USC-HEA037HU
ficolin		SEA786Hu
hemopexin		SEB986HU
gelsolin		SEA372Hu
C4 binding protein alpha		SEB620Hu
retinol binding protein 4		SEA929Hu
talin		SEA278Hu
leucine rich alpha-2-glycoprotein 1		HEB934Hu high sensitive
lumican	RayBiotech	ELH-LUM-1
serum amyloid A		ELH-SAA-1
vitronectin		ELH-VTN
carboxyterminal propeptide of type I procollagen	CUSABIO	CSB-E08079h
fibrinogen gamma chain		CSB-E13319h
alpha-trypsin inhibitor heavy chain H4		CSB-E17022h
hyaluronan-binding protein 2		CSB-EL010113HU
complement component C9		CSB-E14011h
serum amyloid A	Boster	BOS-EK1544, SAA/SAA1 PicoKine Kit
TGF-β1	Biovendor	BVD-RAF122R
adiponectin		BVD-RD191023100

The assays were performed according the suppliers instructions with the following modifications: all assays from Cloud-Clone and CUSBIO were performed with double incubation time to increase sensitivity. Samples were pre-incubated with 0.5% Tween 20 before measured of gelsolin to reach dilutional linearity. For the quantification of PICP, TGF-β1, and CRP in urine either incubation times were prolonged or concentrated specimens were applied. For that samples have been concentrated in Amicon® Ultra -15 Centrifugal filters (3K, UFC900324) by ~10 to ~60 fold depending on the starting volume available.

Supplementary Tables S3 to S8: ROC analysis, comparison of BM values from patient´s groups with healthy controls

Table S3: Patients with obesity, hypertension, metabolic syndrome, and T2DM (group A)

BM	Cut-off	Sensitivity	1-specificity	AUC	BM*BM	Cut-off	Sensitivity	1-specificity	AUC
**serum**									
VTN	40.75	0.636	0.130	0.810					
CRP	19.78	0.765	0.346	0.769					
**urine**									
ColXIII	0.18	0.600	0.300	0.717	ColXIII*FMN	8.07	0.667	0.253	0.771
HABP2	0.19	0.615	0.306	0.726	C4BP*ColXIII	0.35	0.800	0.265	0.763
CRP	5.73	0.714	0.158	0.810	HABP2*FGG	212.9	0.462	0.173	0.736
					C4BP*ColXIII*HABP2	0.10	0.636	0.156	0.766

Table S4: Patients with T1DM (group B)

BM	Cut-off	Sensitivity	1-specificity	AUC	BM*BM	Cut-off	Sensitivity	1-specificity	AUC
**serum**									
PICP	34.65	0.786	0.211	0.838					
C9	5048.0	0.545	0.111	0.758					
**urine**									
TGFβ	13.69	0.659	0.293	0.652	ADP*PICP	44.04	0.651	0.242	0.718
CRP	2.52	0.516	0.253	0.626	HABP2*TGFβ	2.26	0.756	0.167	0.801
CFH	4.29	0.671	0.455	0.676					
CFI	0.70	0.516	0.354	0.605					

Table S5: Patients with renal agenesis, hypoplasia, multicystic renal dysplasia (group C)

BM	Cut-off	Sensitivity	1-specificity	AUC	BM*BM	Cut-off	Sensitivity	1-specificity	AUC
					**urine**				
ColXIII	0.18	0.433	0.3	0.625	ColXIII*C4BP	0.7664	0.536	0.224	0.696
HABP2	0.185	0.778	0.306	0.714					
C4BP	5.53	0.515	0.16	0.685					

Table S6: Patients with anomalies of the urinary tract, duplex kidneys (group D)

BM	Cut-off	Sensitivity	1-specificity	AUC	BM*BM	Cut-off	Sensitivity	1-specificity	AUC
					**serum**				
VTN	40.6	0.643	0.13	0.770					
					**urine**				
ColXIII	0.18	0.611	0.300	0.692	C4BP*ColXIII*HABP2	0.55	0.462	0.115	0.661
HABP2	0.19	0.714	0.306	0.758					

Table S7: Patients with post-infectious GN, IgAN, IgAV (group G)

BM	Cut-off	Sensitivity	1-specificity	AUC	BM*BM	Cut-off	Sensitivity	1-specificity	AUC
					**serum**				
CRP	31.345	0.750	0.231	0.774					
					**urine**				
AGT	1.37	0.857	0.125	0.894	ADP*C4BP	26.9	0.714	0.121	0.870
ColXIII	0.97	0.625	0.130	0.854	AGT*GS	220.5	0.667	0.052	0.872
GS	155.5	0.667	0.071	0.815	C4BP*ColXIII	7.00	0.750	0.092	0.913
LRGP1	1.97	0.625	0.158	0.746	ColXIII*FMN	70.9	0.625	0.101	0.888
VTN	3.27	0.500	0.158	0.730	GS*CFH	3601	0.667	0.111	0.840
C4BP	6.09	0.750	0.150	0.881	GS*VTN	380.2	0.667	0.081	0.847
CRP	4.08	0.750	0.208	0.867	GS*CFI	670.3	0.667	0.010	0.867
CFH	23.73	0.667	0.152	0.813	HABP2*FGG	1103	0.667	0.061	0.854
CFI	1.49	0.857	0.152	0.883	VTN*CFI	2.80	0.857	0.202	0.875
FMN	91.2	0.625	0.150	0.817	AGT*VTN	11.7	0.667	0.010	0.845
FGG	1408	0.875	0.178	0.906	CFH*CFI	116.1	0.667	0.071	0.875
					CFH*FGG	57539	0.667	0.061	0.862
					CFI*FGG	2152	0.857	0.101	0.921
					CFI*a1AGP	43.3	0.714	0.082	0.875
					C4BP*ColXIII*HABP2	6.90	0.667	0.031	0.936

Table S8: Entire patients (group A to G)

BM	Cut-off	Sensitivity	1-specificity	AUC	BM*BM	Cut-off	Sensitivity	1-specificity	AUC
					**serum**				
TGFβ	32.7	0.449	0.05	0.684					
					**urine**				
ColXIII	0.02	0.837	0.38	0.703	ADP*C4BP	25.09	0.396	0.131	0.667
HABP2	0.25	0.586	0.224	0.757	ColXIII*FMN	0.23	0.766	0.330	0.706
C4BP	5.01	0.558	0.17	0.719	ColXIII*C4BP	0.001	0.822	0.381	0.742
CRP	2.52	0.516	0.253	0.626	HABP2*TGFβ	2.10	0.619	0.178	0.704
CFH	4.29	0.671	0.455	0.676	HABP2*FGG	143.4	0.595	0.255	0.744
CFI	0.70	0.516	0.354	0.605	ColXIII*HABP2*C4BP	0.08	0.552	0.156	0.721

Tables S3 to S8:

ROC analysis by SPSS (cf. Methods section). Only BMs with AUC >0.600 are shown.

Concentration ranges of BMs: Serum: ColXII, HABP2, PICP, TGF-β1 and C1q, ng/ml, GS, µg/ml.

Urine: ColXIII, HABP2, C4BP, CFH, CFI, FGG, GS, LRGP1, a1AGP, and C1q, ng/mg creatinine; CRP, C9 and FMN, pg/mg creatinine.

Pearson	ADP	a1AGP	AGT	Col-XIII	GS	LRGP1	HABP2	PICP	TGFß	VTN	C9	C4BP	CRP	CFH	CFI	FMN	LUM	FGG	C1q	Spearman
**ADP**		0.284[b]<br>0.000	0.380[b]<br>0.000	-0.056<br>0.367	0.365[b]<br>0.000	0.271[b]<br>0.000	0.172[b]<br>0.006	0.417[b]<br>0.000	0.210[b]<br>0.001	0.326[b]<br>0.000	0.398[b]<br>0.000	-0.049<br>0.417	-0.077<br>0.207	-0.042<br>0.497	0.281[b]<br>0.000	0.193[b]<br>0.002	-0.244<br>0.251	0.213[b]<br>0.000	0.107<br>0.181	**ADP**
**a1AGP**	0.318[b]<br>0.000		0.410[b]<br>0.000	0.280[b]<br>0.000	0.511[b]<br>0.000	0.149[a]<br>0.014	0.320[b]<br>0.000	0.387[b]<br>0.000	0.043<br>0.500	0.248[b]<br>0.000	**0.652[b]**<br>0.000	0.404[b]<br>0.000	0.201[b]<br>0.001	0.489[b]<br>0.000	**0.703[b]**<br>0.000	0.333[b]<br>0.000	0.425[a]<br>0.038	0.420[b]<br>0.000	0.415[b]<br>0.000	**a1AGP**
**AGT**	0.495[b]<br>0.000	0.260[b]<br>0.000		0.141[a]<br>0.024	**0.746[b]**<br>0.000	0.128[a]<br>0.037	0.492[b]<br>0.000	**0.517[b]**<br>0.000	0.353[b]<br>0.000	**0.694[b]**<br>0.000	**0.570[b]**<br>0.000	0.256[b]<br>0.000	00.098<br>0.116	0.186[b]<br>0.003	**0.663[b]**<br>0.000	0.372[b]<br>0.000	**0.674[b]**<br>0.000	**0.521[b]**<br>0.000	0.342[b]<br>0.000	**AGT**
**Col-XIII**	0.041<br>0.506	0.256[b]<br>0.000	0.327[b]<br>0.000		0.250[b]<br>0.000	0.185[b]<br>0.002	0.380[b]<br>0.000	0.066<br>0.291	-0.178[b]<br>0.005	-0.046<br>0.455	0.133[a]<br>0.032	**0.527[b]**<br>0.000	0.317[b]<br>0.000	**0.529[b]**<br>0.000	0.313[b]<br>0.000	0.299[b]<br>0.000	0.290<br>0.170	0.363[b]<br>0.000	0.290[b]<br>0.000	**Col-XIII**
**GS**	0.493[b]<br>0.000	0.221[b]<br>0.000	**0.702[b]**<br>0.000	0.212[b]<br>0.001		0.117<br>0.062	0.422[b]<br>0.000	0.409[b]<br>0.000	0.161[a]<br>0.014	0.478[b]<br>0.000	**0.603[b]**<br>0.000	0.315[b]<br>0.000	0.123<br>0.052	0.343[b]<br>0.000	**0.751[b]**<br>0.000	0.402[b]<br>0.000	0.484[a]<br>0.016	0.467[b]<br>0.000	0.383[b]<br>0.000	**GS**
**LRGP1**	0.251[b]<br>0.000	0.095<br>0.119	0.258[b]<br>0.000	0.108<br>0.079	0.088<br>0.159		0.154[a]<br>0.014	0.091<br>0.134	0.045<br>0.482	0.047<br>0.437	0.098<br>0.111	0.330[b]<br>0.000	0.243[b]<br>0.000	0.228[b]<br>0.000	0.177[b]<br>0.004	0.354[b]<br>0.000	0.431[a]<br>0.035	0.336[b]<br>0.000	0.350[b]<br>0.000	**LRGP1**
**HABP2**	0.078<br>0.217	0.384[b]<br>0.000	0.242[b]<br>0.000	0.310[b]<br>0.000	0.128[a]<br>0.040	0.041<br>0.511		0.290[b]<br>0.000	-0.049<br>0.459	0.258[b]<br>0.000	0.337[b]<br>0.000	0.367[b]<br>0.000	0.228[b]<br>0.000	0.326[b]<br>0.000	0.458[b]<br>0.000	0.364[b]<br>0.000	**0.614[b]**<br>0.001	0.374[b]<br>0.000	0.176[a]<br>0.028	**HABP2**
**PICP**	**0.584[b]**<br>0.000	0.425[b]<br>0.000	0.390[b]<br>0.000	0.081<br>0.193	0.441[b]<br>0.000	0.078<br>0.199	0.233[b]<br>0.000		**0.511[b]**<br>0.000	**0.561[b]**<br>0.000	0.467[b]<br>0.000	0.109<br>0.076	-0.032<br>0.590	0.121<br>0.051	0.493[b]<br>0.000	0.063<br>0.307	0.144<br>0.511	0.325[b]<br>0.000	0.267[b]<br>0.001	**PICP**
**TGFβ**	0.039<br>0.542	0.144[a]<br>0.024	0.030<br>0.645	0.070<br>0.274	0.018<br>0.780	-0.019<br>0.764	**0.586[b]**<br>0.000	0.230[b]<br>0.000		0.460[b]<br>0.000	0.263[b]<br>0.000	-0.155[a]<br>0.015	-0.132[a]<br>0.039	-0.088<br>0.176	0.167[b]<br>0.010	0.042<br>0.511	0.060<br>0.779	0.162[a]<br>0.011	0.232[b]<br>0.006	**TGFβ**
**VTN**	0.416[b]<br>0.000	0.463[b]<br>0.000	**0.575[b]**<br>0.000	0.046<br>0.451	**0.553[b]**<br>0.000	0.259[b]<br>0.000	0.162[b]<br>0.009	0.456[b]<br>0.000	0.010<br>0.870		**0.519[b]**<br>0.000	0.127[a]<br>0.036	-0.007<br>0.914	-0.030<br>0.627	0.459[b]<br>0.000	0.112<br>0.069	0.436[a]<br>0.033	0.405[b]<br>0.000	0.224[b]<br>0.005	**VTN**
**C9**	**0.560[b]**<br>0.000	0.456[b]<br>0.000	**0.563[b]**<br>0.000	0.093<br>0.134	**0.531[b]**<br>0.000	0.271[b]<br>0.000	0.229[b]<br>0.000	0.398[b]<br>0.000	0.028<br>0.669	**0.626[b]**<br>0.000		0.240[b]<br>0.000	0.167[b]<br>0.007	0.358[b]<br>0.000	**0.704[b]**<br>0.000	0.270[b]<br>0.000	**0.535[b]**<br>0.007	0.372[b]<br>0.000	**0.502[b]**<br>0.000	**C9**
**C4BP**	**0.548[b]**<br>0.000	0.370[b]<br>0.000	0.278[b]<br>0.000	0.263[b]<br>0.000	0.336[b]<br>0.000	0.149[a]<br>0.014	0.267[b]<br>0.000	0.315[b]<br>0.000	0.003<br>0.965	0.247[b]<br>0.000	0.490[b]<br>0.000		0.359[b]<br>0.000	**0.577[b]**<br>0.000	0.492[b]<br>0.000	0.342[b]<br>0.000	**0.713[b]**<br>0.000	0.437[b]<br>0.000	0.291[b]<br>0.000	**C4BP**
**CRP**	-0.041<br>0.496	0.034<br>0.585	0.024<br>0.701	0.397[b]<br>0.000	0.039<br>0.536	0.214[b]<br>0.00	0.066<br>0.298	-0.033<br>0.581	-0.011<br>0.858	-0.016<br>0.788	0.001<br>0.983	0.123[a]<br>0.044		0.309[b]<br>0.000	0.272[b]<br>0.000	0.255[b]<br>0.000	**0.574[b]**<br>0.003	0.244[b]<br>0.000	0.272[b]<br>0.001	**CRP**
**CFH**	0.411[b]<br>0.000	0.237[b]<br>0.000	0.545[b]<br>0.000	0.252[b]<br>0.000	**0.747[b]**<br>0.000	0.173[b]<br>0.005	0.352[b]<br>0.000	0.315[b]<br>0.000	0.010<br>0.872	0.486[b]<br>0.000	**0.560[b]**<br>0.000	0.444[b]<br>0.000	0.101<br>0.104		0.490[b]<br>0.000	0.339[b]<br>0.000	0.460[a]<br>0.024	0.306[b]<br>0.000	0.393[b]<br>0.000	**CFH**
**CFI**	0.409[b]<br>0.000	0.596[b]<br>0.00	0.484[b]<br>0.000	0.229[b]<br>0.000	**0.686[b]**<br>0.000	0.082<br>0.182	0.467[b]<br>0.000	0.446[b]<br>0.000	0.029<br>0.655	**0.601[b]**<br>0.000	0.486[b]<br>0.000	0.474[b]<br>0.000	0.069<br>0.268	**0.740[b]**<br>0.000		0.367[b]<br>0.000	**0.516[b]**<br>0.010	0.497[b]<br>0.000	**0.545[b]**<br>0.000	**CFI**
**FMN**	0.122[a]<br>0.047	0.383[b]<br>0.000	0.371[b]<br>0.000	**0.569[b]**<br>0.000	0.138[a]<br>0.030	0.096<br>0.116	0.464[b]<br>0.000	0.140[a]<br>0.022	0.011<br>0.867	0.094<br>0.129	0.178[b]<br>0.004	0.388[b]<br>0.000	0.167[b]<br>0.006	0.330[b]<br>0.000	0.314[b]<br>0.000		**0.772[b]**<br>0.000	0.346[b]<br>0.000	0.352[b]<br>0.000	**FMN**
**LUM**	-0.086<br>0.689	0.230<br>0.279	0.505[a]<br>0.012	0.121<br>0.572	0.152<br>0.477	0.087<br>0.686	0.241<br>0.256	-0.093<br>0.671	0.292<br>0.166	0.156<br>0.465	0.132<br>0.537	0.475[a]<br>0.019	0.026<br>0.903	0.191<br>0.372	0.116<br>0.589	**0.633[b]**<br>0.002		**0.569[b]**<br>0.006	0.317<br>0.141	**LUM**
**FGG**	0.176[b]<br>0.004	0.343[b]<br>0.000	0.401[b]<br>0.000	0.452[b]<br>0.000	0.284[b]<br>0.000	0.154[a]<br>0.011	**0.600[b]**<br>0.000	0.192[b]<br>0.002	0.019<br>0.770	0.323[b]<br>0.000	0.353[b]<br>0.000	0.397[b]<br>0.000	0.201[b]<br>0.001	**0.527[b]**<br>0.000	**0.560[b]**<br>0.000	**0.730[b]**<br>0.000	0.411<br>0.057		0.396[b]<br>0.000	**FGG**
**C1q**	-0.024<br>0.766	0.020<br>0.801	-0.030<br>0.710	0.318[b]<br>0.000	0.022<br>0.783	**0.694[b]**<br>0.000	0.035<br>0.665	0.005<br>0.955	0.017<br>0.841	0.021<br>0.795	0.041<br>0.611	0.215[b]<br>0.007	**0.558[b]**<br>0.000	0.456[b]<br>0.000	0.124<br>0.121	0.073<br>0.369	-0.124<br>0.575	0.089<br>0.268		**C1q**

Supplementary Table S2: Correlation of each BM in urine with each other's.

Analyses were performed by SPSS (v.27). Lower left halve: correlation according Pearson; upper right halve: correlation according Spearman. Each cell shows the correlation coefficient (upper figure) and a p-value (lower figure).

[a] and [b] indicate significant and highly significant correlations proposed by SPSS. Notwithstanding that, we set a correlation coefficient >0.500 moderately significant and >0.600 highly significant and mark these values by blue (Pearson) and green (Spearman) filling.

Supplementary Table S9 to S11:

Sub-group of healthy controls

Table S9: Correlation of serum and urinary BM candidates with BMI z-score

	BMI z-score					
**BM in →**	**Serum**			**Urine**		
**↓**	Pearson	Spearm.	**n**	Pearson	Spearm.	**n**
**ADP**	-0.358<br>0.253	-0.417<br>0.178	12	0.077<br>0.447	0.059<br>0.561	99
**a1AGP**	-0.188<br>0.502	-0.104<br>0.713	15	-0.027<br>0.792	-0.092<br>0.370	98
**AGT**	0.005<br>0.988	-0.004<br>0.991	12	0.125<br>0.23	0.085<br>0.415	94
**ColXIII**	0.255<br>0.424	0.392<br>0.207	12	-0.045<br>0.659	0.044<br>0.664	99
**GS**	0.150<br>0.641	0.067<br>0.837	12	0.077<br>0.454	-0.034<br>0.742	97
**LRGP1**	-0.159<br>0.467	-0.184<br>0.401	23	0.199[a]<br>0.049	-0.029<br>0.779	99
**HABP2**	-0.075<br>0.817	-0.049<br>0.880	12	-0.031<br>0.762	0.002<br>0.981	96
**PICP**	0.136<br>0.590	-0.033<br>0.896	18	-0.050<br>0.624	-0.119<br>0.240	99
**TGFβ**	-0.318<br>0.198	-0.306<br>0.217	18	-0.027<br>0.802	-0.158<br>0.132	92
**VTN**	-0.087<br>0.693	-0.097<br>0.674	23	0.070<br>0.488	-0.041<br>0.683	99
**C9**	-0.061<br>0.897	-0.321<br>0.482	7	-0.090<br>0.383	-0.071<br>0.491	97
**C4BP**	-0.132<br>0.684	-0.319<br>0.313	12	-0.085<br>0.405	-0.201[a]<br>0.047	99
**CRP**	-0.230<br>0.279	-0.118<br>0.583	24	0.014<br>0.890	0.071<br>0.484	100
**FMN**	0.065<br>0.840	0.189<br>0.556	12	-0.043<br>0.672	-0.072<br>0.484	99
**CFH**			3	-0.043<br>0.678	-0.084<br>0.416	98
**CFI**	-0.053<br>0.910	-0.286<br>0.535	7	-0.094<br>0.361	-0.163<br>0.111	98
**FGG**	-0.271<br>0.349	-0.495<br>0.072	14	0.035<br>0.731	0.027<br>0.794	99
**C1q**	-0.190<br>0.576	-0.305<br>0.361	11	-0.227[a]<br>0.035	-0.235[a]<br>0.030	86

Analyses were performed by SPSS (v.27). Each cell shows the correlation coefficient (upper figure) and a p-value (lower figure).

[a] and [b] indicate significant and highly significant correlations proposed by SPSS. Notwithstanding that, we set a correlation coefficient >0.500 moderately significant and >0.600 highly significant and mark these values by grey filling.

Table S10: Correlation of serum and urinary BM candidates with age

	Age [y]					
BM in →	Serum			Urine		
↓	Pearson	Spearm.	n	Pearson	Spearm.	n
ADP	-0.264	-0.343	12	-0.249[a]	-0.227[a]	101
	0.408	0.276		0.012	0.023	
a1AGP	-0.311	-0.323	17	0.027	-0.123	100
	0.224	0.206		0.787	0.222	
AGT	0.858[b]	0.860[b]	12	-0.274[b]	-0.230[a]	96
	<0.001	<0.001		0.007	0.024	
ColXIII	-0.487	-0.49	12	0.183	0.364[b]	100
	0.108	0.106		0.068	<0.001	
GS	0.429	0.483	12	-0.293[b]	-0.174	99
	0.164	0.112		0.003	0.085	
LRGP1	0.173	0.142	25	-0.151	-0.191	101
	0.408	0.497		0.132	0.056	
HABP2	0.314	0.364	12	0.176	-0.021	98
	0.320	0.245		0.084	0.839	
PICP	0.461[a]	0.318	20	-0.098	-0.010	101
	0.041	0.172		0.332	0.924	
TGFβ	0.368	0.366	20	-0.213[a]	-0.276[b]	93
	0.111	0.112		0.040	0.007	
VTN	0.243	0.280	23	-0.289[b]	-0.334[b]	101
	0.265	0.195		0.003	<0.001	
C9	0.583	0.583	9	-0.132	-0.241[a]	99
	0.100	0.099		0.194	0.016	
C4BP	0.075	0.217	12	0.040	0.030	100
	0.817	0.499		0.695	0.765	
CRP	-0.331	-0.039	26	-0.022	0.013	101
	0.098	0.850		0.824	0.898	
FMN	0.073	-0.027	13	-0.238[a]	-0.102	100
	0.814	0.929		0.017	0.311	
CFH			3	0.058	0.062	99
				0.571	0.539	
CFI	0.165	0.333	9	-0.095	-0.155	99
	0.672	0.381		0.351	0.125	
FGG	-0.149	-0.160	14	-0.127	-0.151	101
	0.612	0.584		0.205	0.133	
C1q	0.888[b]	0.791[b]	11	-0.150	-0.224[a]	86
	<0.001	0.004		0.168	0.038	

Graphical visualization of correlations considered significant

Serum angiotensinogen and age

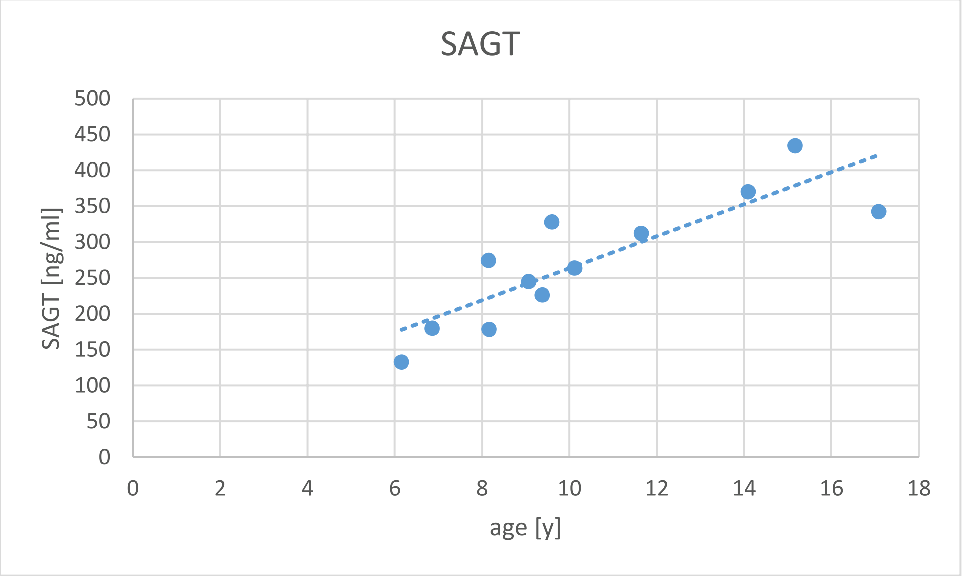

Serum complement component C1q and age

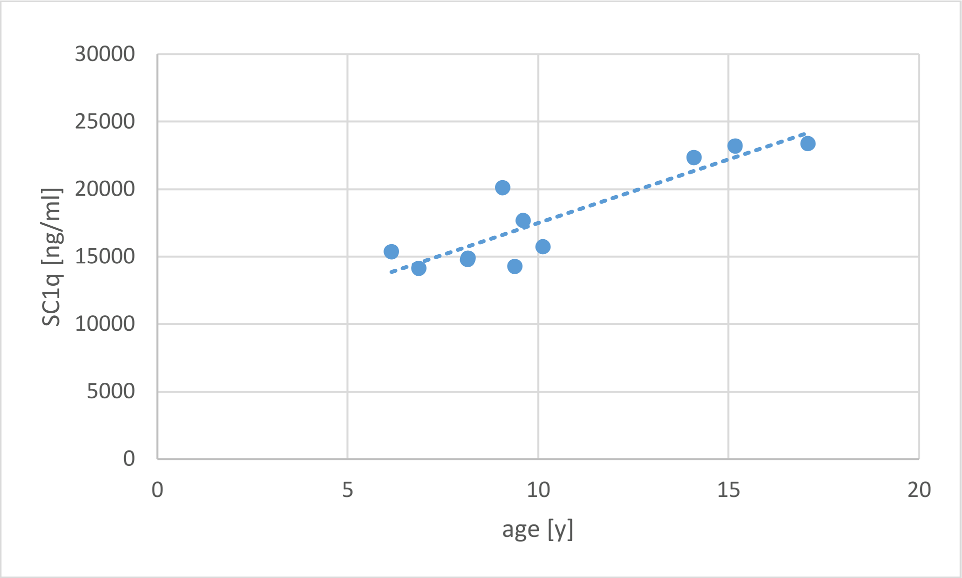

Table S11: Correlation of serum and urinary BM candidates with blood pressure z-score

BM in → ↓	Systolic blood pressure z-score						Diastolic blood pressure z-score					
	Serum			Urine			Serum			Urine		
	Pears.	Spearm.	**n**	Pears.	Spearm.	**n**	Pears.	Spearm.	**n**	Pears.	Spearm.	**n**
**ADP**	-0.312	-0.255	10	0.081	-0.008	97	-0.883[b]	-0.770[b]	10	0.040	0.001	97
	0.380	0.476		0.431	0.937		<0.001	0.009		0.695	0.993	
**a1AGP**	0.276	0.481	15	0.202[a]	0.091	96	-0.116	-0.011	15	0.202[a]	0.106	96
	0.319	0.069		0.048	0.377		0.680	0.970		0.048	0.302	
**AGT**	0.452	0.401	10	00.248[a]	0.217[a]	92	0.323	0.224	10	0.245[a]	0.235[a]	92
	0.189	0.250		0.017	0.038		0.362	0.533		0.019	0.024	
**ColXIII**	-0.363	-0.413	10	0.011	0.108	97	0.208	0.006	10	0.029	0.065	97
	0.303	0.235		0.913	0.292		0.565	0.987		0.779	0.525	
**GS**	0.605	0.596	10	-0.074	-0.022	95	0.325	0.297	10	0.101	0.041	95
	0.064	0.069		0.476	0.834		0.359	0.405		0.331	0.69	
**LRGP1**	-0.214	-0.382	21	0.097	0.045	97	0.031	-0.212	21	0.087	0.112	97
	0.352	0.087		0.344	0.659		0.893	0.355		0.397	0.277	
**HABP2**	0.259	0.419	10	0.352[b]	0.122	94	-0.600	-0.648[a]	10	0.219[a]	0.189	94
	0.470	0.228		<0.001	0.240		0.066	0.043		0.034	0.068	
**PICP**	-0.190	-0.187	16	0.061	0.098	97	-0.392	-0.476	16	0.136	0.189	97
	0.481	0.488		0.553	0.338		0.133	0.062		0.186	0.064	
**TGFβ**	0.073	0.178	16	0.121	0.082	92	-0.047	-0.038	16	0.041	0.093	92
	0.787	0.509		0.250	0.438		0.863	0.888		0.699	0.377	
**VTN**	-0.002	-0.004	19	0.016	0.171	97	-0.184	-0.140	19	0.185	0.235[a]	97
	0.994	0.986		0.879	0.094		0.451	0.567		0.07	0.021	
**C9**	0.154	0.036	7	-0.123	-0.081	95	-0.499	-0.429	7	-0.071	-0.047	95
	0.742	0.939		0.235	0.434		0.255	0.337		0.492	0.652	
**C4BP**	0.401	0.231	10	0.234[a]	-0.015	96	0.159	0.055	10	0.324[b]	0.170	96
	0.251	0.521		0.022	0.887		0.661	0.881		0.001	0.098	
**CRP**	-0.266	-0.142	22	-0.030	-0.060	97	-0.217	-0.277	22	0.019	-0.041	97
	0.231	0.529		0.771	0.557		0.333	0.212		0.85	0.689	
**FMN**	0.118	0.164	10	-0.004	-0.002	96	0.069	-0.164	10	0.002	-0.014	96
	0.745	0.650		0.973	0.982		0.850	0.651		0.984	0.891	
**CFH**			3	0.056	0.040	95			3	0.134	0.117	95
				0.588	0.701					0.196	0.261	
**CFI**	0.643	0.714	7	0.128	0.055	95	0.341	0.143	7	0.208[a]	0.128	95
	0.120	0.071		0.218	0.594		0.454	0.760		0.044	0.215	
**FGG**	-0.059	-0.042	12	0.183	0.123	97	-0.227	-0.126	12	0.212[a]	0.147	97
	0.855	0.897		0.074	0.231		0.478	0.697		0.037	0.150	
**C1q**	0.613	0.444	9	-0.139	-0.075	83	0.053	-0.133	9	-0.122	-0.102	83
	0.079	0.232		0.211	0.502		0.892	0.732		0.272	0.360	

Graphical visualization of the correlation considered significant

Serum adiponectin and diastolic blood pressure.

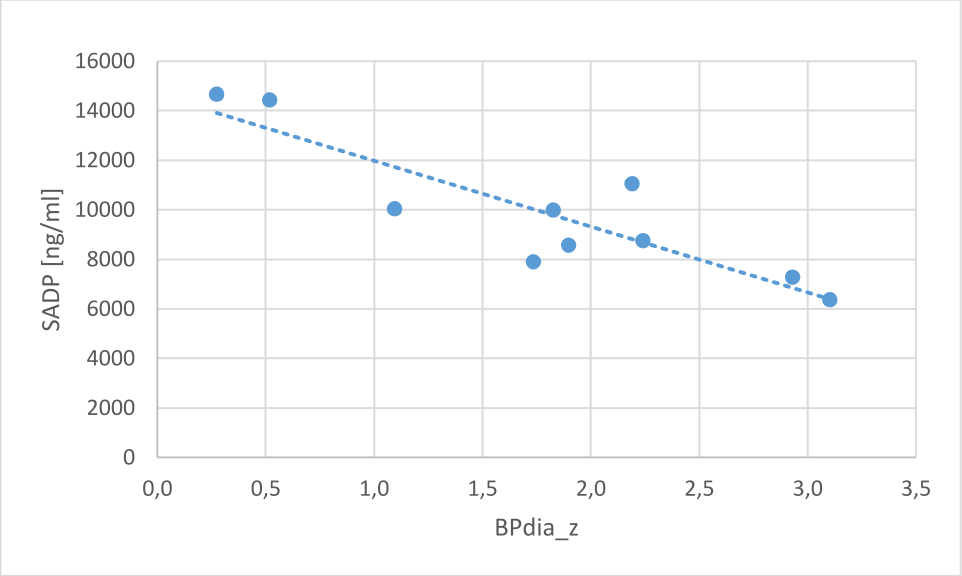

Supplementary Tables S12 to S15:

Patients with obesity, hypertension, metabolic syndrome, and T2DM (group A)

Table S12: Correlation of serum and urinary BM candidates with UACR and blood glucose

	**UACR**						**blood glucose**					
**BM in→**	**Serum**			**Urine**			**Serum**			**Urine**		
**↓**	**Pearson**	**Spearman**	**n**	**Pearson**	**Spearman**	**n**	**Pearson**	**Spearman**	**n**	**Pearson**	**Spearman**	**n**
**ADP**			0	-0.046<br>0.881	0.110<br>0.720	13			0	0.120<br>0.710	0.088<br>0.787	12
**a1AGP**	-0.003<br>0.993	-0.088<br>0.808	10	0.049<br>0.874	0.490<br>0.089	13	-0.546<br>0.102	-0.576<br>0.081	10	0.271<br>0.394	0.046<br>0.888	12
**AGT**			0	-0.240<br>0.429	-0.063<br>0.837	13			0	0.819[b]<br>0.001	0.722[b]<br>0.008	12
**ColXIII**			0	-0.031<br>0.932	0.381<br>0.277	10			0	-0.289<br>0.450	-0.319<br>0.402	9
**GS**			0	-0.043<br>0.889	0.157<br>0.608	13			0	0.700[a]<br>0.011	0.623[a]<br>0.030	12
**LRGP1**	-0.172<br>0.523	-0.109<br>0.688	16	0.098<br>0.749	0.374<br>0.207	13	-0.016<br>0.956	-0.084<br>0.766	15	-0.135<br>0.677	-0.145<br>0.653	12
**HABP2**			0	-0.149<br>0.627	0.133<br>0.665	13			0	0.027<br>0.933	-0.141<br>0.662	12
**PICP**	0.437<br>0.327	0.450<br>0.310	7	0.073<br>0.803	0.526<br>0.053	14	0.227<br>0.624	0.018<br>0.969	7	0.397<br>0.179	-0.011<br>0.971	13
**TGFβ**			0	0.143<br>0.674	0.304<br>0.364	11	-0.329<br>0.471	-0.360<br>0.427	7	0.148<br>0.682	0.204<br>0.571	10
**VTN**	-0.300<br>0.370	-0.656[a]<br>0.028	11	-0.189<br>0.536	-0.025<br>0.936	13	0.160<br>0.658	0.128<br>0.725	10	0.934[b]<br><0.001	0.760[b]<br>0.004	12
**C9**	-0.620<br>0.137	-0.847[a]<br>0.016	7	-0.039<br>0.900	0.353<br>0.237	13	0.081<br>0.863	0.234<br>0.613	7	0.664[a]<br>0.019	0.560<br>0.058	12
**C4BP**			0	-0.498<br>0.083	-0.383<br>0.197	13			0	0.304<br>0.337	0.483<br>0.111	12
**CRP**	-0.509[a]<br>0.037	-0.628[b]<br>0.007	17	-0.467<br>0.093	-0.599[a]<br>0.024	14	0.084<br>0.758	0.109<br>0.688	16	-0.156<br>0.610	-0.063<br>0.837	13
**FMN**			0	-0.277<br>0.384	-0.165<br>0.609	12			3	-0.048<br>0.890	-0.191<br>0.574	11
**CFH**			0	-0.034<br>0.912	0.328<br>0.274	13			0	0.403<br>0.194	0.158<br>0.625	12
**CFI**	0.090<br>0.848	0.414<br>0.355	7	0.051<br>0.868	0.486<br>0.092	13	-0.660<br>0.107	-0.144<br>0.758	7	0.461<br>0.132	0.258<br>0.417	12
**FGG**			0	-0.075<br>0.808	0.240<br>0.430	13			0	0.438<br>0.154	0.445<br>0.147	12

Analyses were performed by SPSS (v.27). Each cell shows the correlation coefficient (upper figure) and a p-value (lower figure).

[a] and [b] indicate significant and highly significant correlations proposed by SPSS. Notwithstanding that, we set a correlation coefficient >0.500 moderately significant and >0.600 highly significant and mark these values by grey filling.

Graphical visualization of correlations considered as significant:

Urinary angiotensinogen (AGT), gelsolin (GS), vitronectin (VTN), and complement component C9 (C9) with blood glucose concentration.

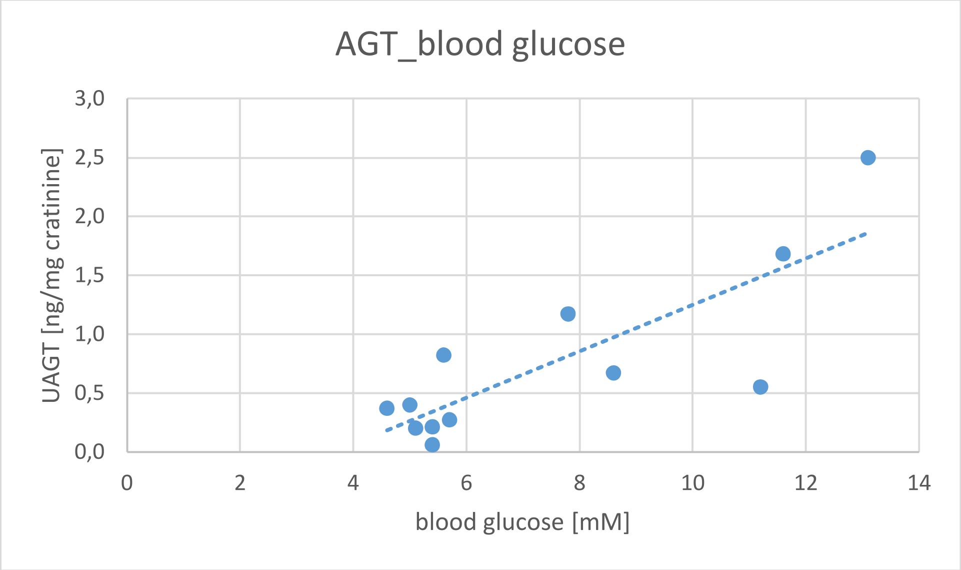

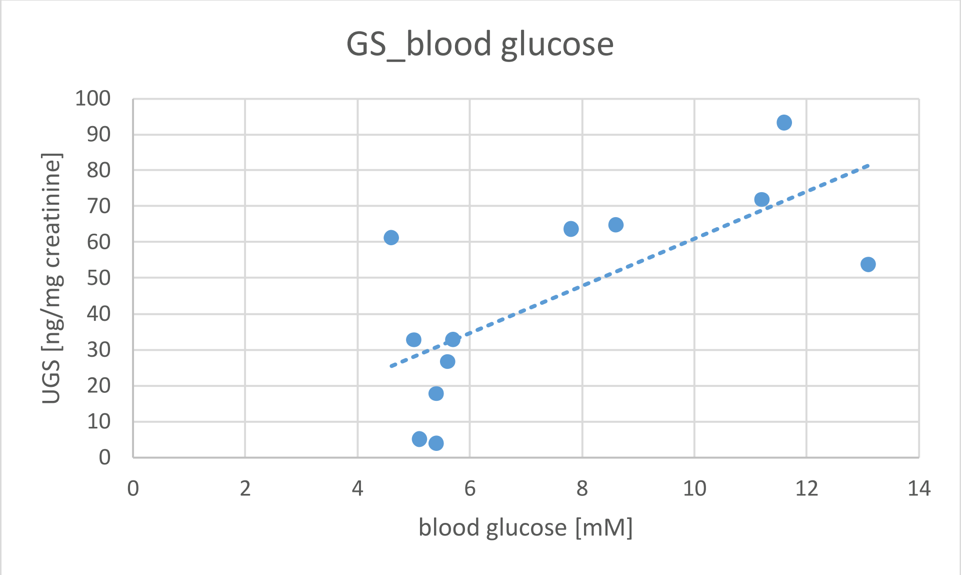

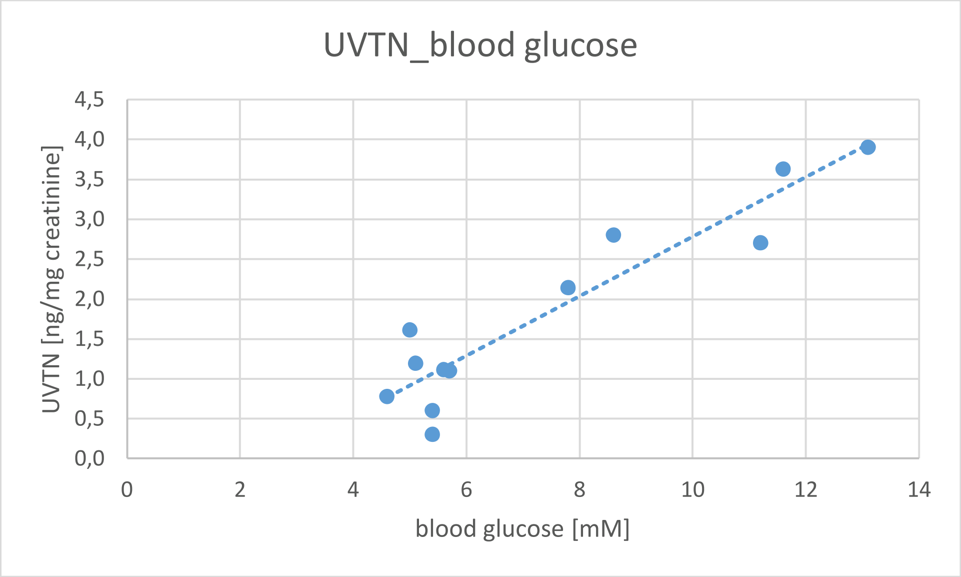

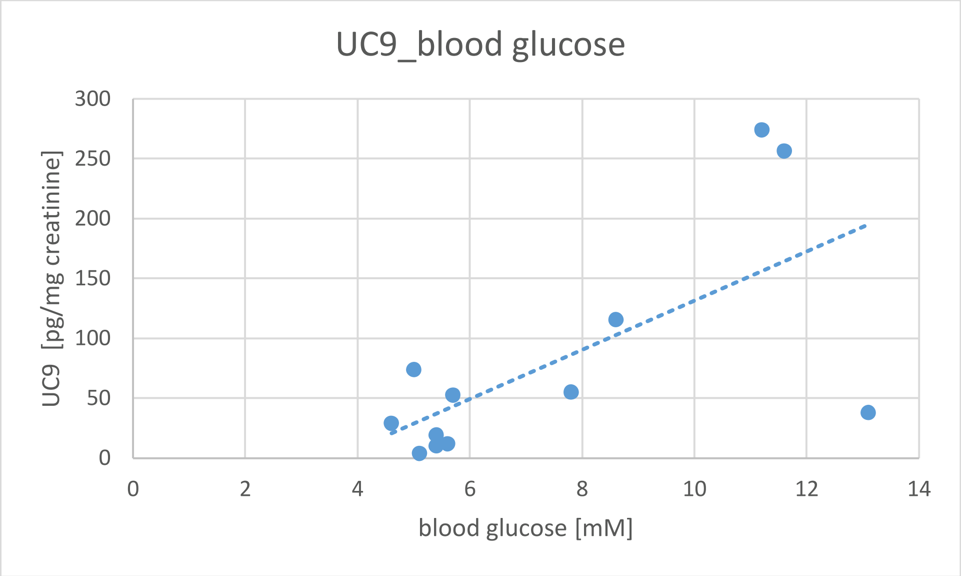
UC9_blood glucose
UC9 [pg/mg creatinine]
300
250
200
150
100
50
0
0
2
4
6
8
10
12
14
blood glucose [mM]

Table S13: Correlation of serum and urinary BM candidates with HbA1c and BMI

	HbA1c						BMI-z-score					
**BM in→**	**Serum**			**Urine**			**Serum**			**Urine**		
**↓**	**Pearson**	**Spearman**	**n**	**Pearson**	**Spearman**	**n**	**Pearson**	**Spearman**	**n**	**Pearson**	**Spearman**	**n**
**ADP**			1	-0.035<br>0.913	-0.077<br>0.812	12			1	0.145<br>0.671	0.318<br>0.340	11
**a1AGP**	0.084<br>0.818	-0.107<br>0.769	10	-0.126<br>0.696	-0.004<br>0.991	12	0.311<br>0.497	0.214<br>0.645	7	0.071<br>0.836	0.273<br>0.417	11
**AGT**			1	-0.284<br>0.371	-0.291<br>0.358	12			1	-0.159<br>0.640	0.036<br>0.915	11
**ColXIII**			1	0.264<br>0.460	0.138<br>0.705	10			1	0.339<br>0.412	0.619<br>0.102	8
**GS**			1	-0.275<br>0.388	-0.175<br>0.585	12			1	-0.018<br>0.959	0.018<br>0.958	11
**LRGP1**	-0.023<br>0.936	-0.167<br>0.552	15	0.112<br>0.730	0.152<br>0.637	12	0.262<br>0.388	0.374<br>0.209	13	-0.091<br>0.790	-0.050<br>0.884	11
**HABP2**			1	-0.308<br>0.330	-0.172<br>0.593	12			1	-0.330<br>0.321	-0.138<br>0.686	11
**PICP**	-0.191<br>0.682	-0.286<br>0.535	7	-0.202<br>0.509	-0.107<br>0.727	13	0.645<br>0.118	0.464<br>0.294	7	0.084<br>0.794	0.070<br>0.829	12
**TGFβ**	0.043<br>0.928	0.071<br>0.879	7	0.515<br>0.127	0.315<br>0.375	10	-0.521<br>0.231	-0.179<br>0.702	7	-0.095<br>0.807	-0.083<br>0.831	9
**VTN**	-0.285<br>0.396	-0.317<br>0.342	11	-0.287<br>0.366	-0.221<br>0.490	12	0.138<br>0.704	0.176<br>0.627	10	-0.066<br>0.847	0.155<br>0.650	11
**C9**	0.407<br>0.365	0.036<br>0.939	7	-0.306<br>0.334	-0.295<br>0.352	12	0.128<br>0.785	0.107<br>0.819	7	0.021<br>0.952	-0.036<br>0.915	11
**C4BP**			1	-0.040<br>0.901	0.014<br>0.965	12			1	0.269<br>0.424	0.300<br>0.370	11
**CRP**	0.503[a]<br>0.047	0.251<br>0.348	16	0.156<br>0.612	0.165<br>0.589	13	0.133<br>0.650	0.095<br>0.748	14	0.422<br>0.172	0.245<br>0.443	12
**FMN**			3	0.010<br>0.977	0.205<br>0.544	11			3	-0.401<br>0.251	-0.164<br>0.651	10
**CFH**			1	-0.184<br>0.568	-0.228<br>0.476	12			1	0.095<br>0.781	0.218<br>0.519	11
**CFI**	0.252<br>0.585	0.036<br>0.939	7	-0.243<br>0.446	-0.255<br>0.424	12	-0.023<br>0.961	-0.143<br>0.760	7	0.117<br>0.731	0.339<br>0.307	11
**FGG**			0	-0.087<br>0.788	0.228<br>0.476	12			0	-0.090<br>0.793	-0.127<br>0.709	11

Table S14: Correlation of serum and urinary BM candidates with serum and urinary CRP

BM in→	SCRP						UCRP					
	Serum			Urine			Serum			Urine		
↓	Pearson	Spearman	n	Pearson	Spearman	n	Pearson	Spearman	n	Pearson	Spearman	n
**ADP**			1	0.318<br>0.314	0.538<br>0.071	12			1	0.152<br>0.621	0.319<br>0.289	13
**a1AGP**	-0.090<br>0.806	0.267<br>0.455	10	-0.021<br>0.949	-0.175<br>0.587	12	-0.436<br>0.280	-0.204<br>0.629	8	0.060<br>0.845	-0.011<br>0.972	13
**AGT**			1	-0.345<br>0.273	-0.315<br>0.319	12			1	-0.008<br>0.979	-0.066<br>0.831	13
**ColXIII**			1	-0.145<br>0.690	-0.067<br>0.854	10			1	-0.163<br>0.653	-0.274<br>0.444	10
**GS**			1	-0.655[a]<br>0.021	-0.448<br>0.145	12			1	-0.285<br>0.345	-0.198<br>0.517	13
**LRGP1**	0.006<br>0.982	0.050<br>0.854	16	0.363<br>0.247	0.169<br>0.599	12			12	0.003<br>0.993	0.163<br>0.594	13
**HABP2**			1	-0.058<br>0.858	0.261<br>0.413	13	0.210<br>0.513	-0.042<br>0.897	1	-0.077<br>0.803	0.064<br>0.835	13
**PICP**	-0.578<br>0.174	-0.679<br>0.094	7	-0.204<br>0.503	-0.236<br>0.437	14	0.149<br>0.750	-0.143<br>0.760	7	0.042<br>0.888	-0.090<br>0.759	14
**TGFβ**	0.453<br>0.308	0.357<br>0.432	7	-0.312<br>0.380	-0.432<br>0.213	10	-0.225<br>0.627	-0.250<br>0.589	7	-0.057<br>0.869	-0.246<br>0.466	11
**VTN**	0.241<br>0.476	0.155<br>0.650	11	-0.417<br>0.178	-0.266<br>0.404	12	0.341<br>0.334	0.127<br>0.726	10	-0.197<br>0.520	-0.236<br>0.437	13
**C9**	0.585<br>0.168	0.357<br>0.432	7	-0.398<br>0.200	-0.462<br>0.131	12	0.317<br>0.489	0.464<br>0.294	7	-0.179<br>0.559	-0.352<br>0.239	13
**C4BP**			1	0.426<br>0.168	0.315<br>0.319	12			1	0.248<br>0.414	0.159<br>0.603	13
**CRP**	1.000	1.000<br>.	17	0.645[a]<br>0.017	0.753[b]<br>0.003	13	0.645[a]<br>0.017	0.753[b]<br>0.003	13	1.000	1.000<br>.	13
**LUM**			0			0			0			0
**FMN**			3	0.192<br>0.572	0.355<br>0.285	11			3	-0.128<br>0.693	0.161<br>0.618	12
**CFH**			1	-0.202<br>0.528	-0.413<br>0.183	12			1	0.101<br>0.742	-0.308<br>0.306	13
**CFI**	0.356<br>0.433	0.071<br>0.879	7	-0.255<br>0.423	-0.526<br>0.079	12	0.116<br>0.805	-0.107<br>0.819	7	0.084<br>0.785	-0.404<br>0.170	13
**FGG**			0	0.064<br>0.843	0.056<br>0.863	12			0	0.017<br>0.956	0.093<br>0.762	13
**C1q**			0			2			0			2

Graphical visualization of correlations considered as significant:

Urinary gelsolin (GS) with serum CRP

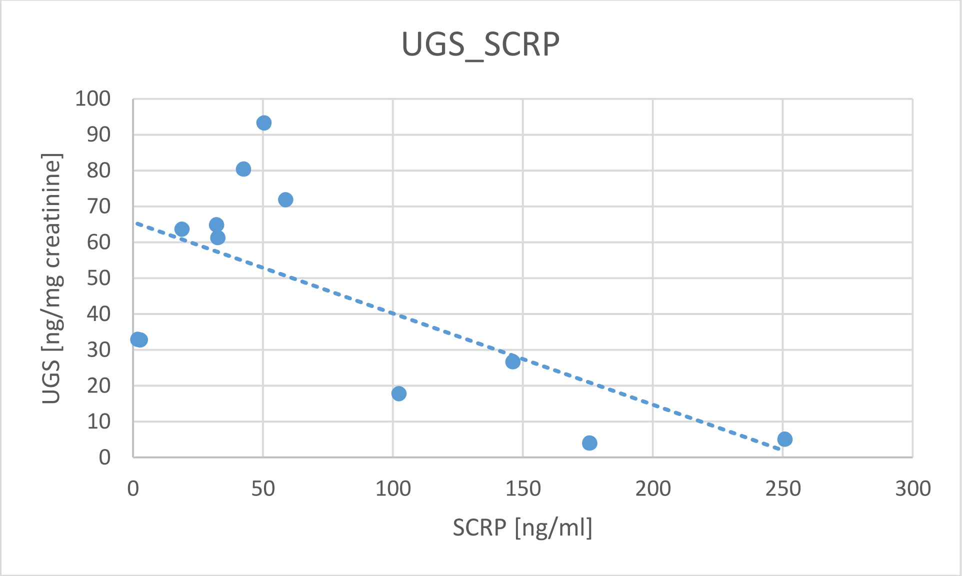

Urinary CRP with serum CRP

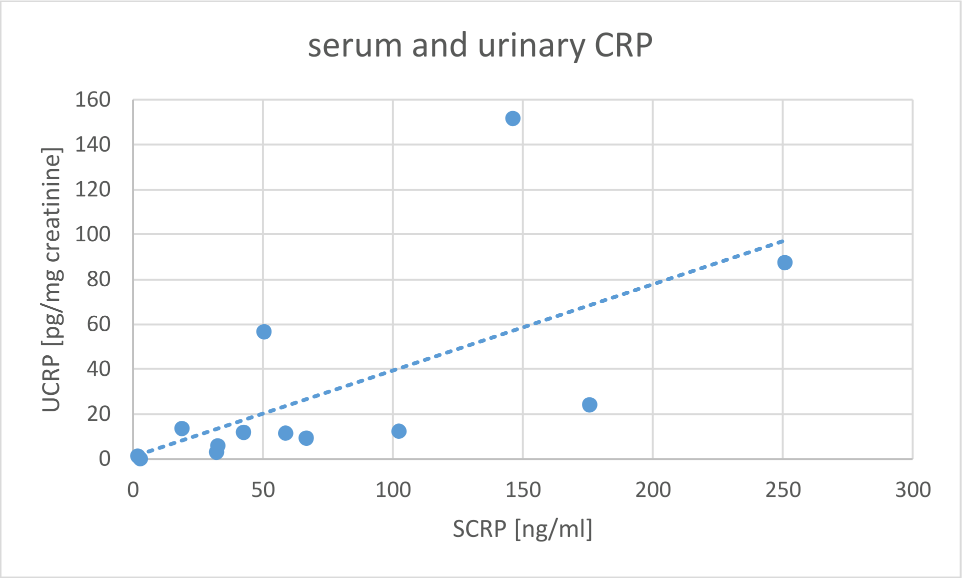

Table S15: Correlation of serum and urinary BM candidates with blood pressure (BP z-score)

BM in→	BP_sys_z-score						BP_dia_z-score					
	Serum			Urine			Serum			Urine		
↓	Pearson	Spearman	n	Pearson	Spearman	n	Pearson	Spearman	n	Pearson	Spearman	n
**ADP**			1	0.753[a]<br>0.012	0.867[b]<br>0.001	10			1	-0.292<br>0.414	-0.383<br>0.275	10
**a1AGP**	0.353<br>0.492	0.543<br>0.266	6	0.537<br>0.110	0.285<br>0.425	10	-0.296<br>0.569	-0.143<br>0.787	6	-0.231<br>0.521	-0.103<br>0.776	10
**AGT**			1	0.339<br>0.338	0.261<br>0.467	10			1	0.098<br>0.787	0.073<br>0.841	10
**ColXIII**			1	0.328<br>0.473	0.500<br>0.253	7			1	0.433<br>0.332	0.179<br>0.702	7
**GS**			1	0.067<br>0.855	-0.067<br>0.855	10			1	0.573<br>0.084	0.480<br>0.160	10
**LRGP1**	-0.029<br>0.929	0.035<br>0.914	12	0.480<br>0.161	-0.170<br>0.638	10	-0.323<br>0.307	-0.434<br>0.158	12	-0.078<br>0.829	0.061<br>0.867	10
**HABP2**			1	-0.268<br>0.454	0.006<br>0.987	10			1	-0.021<br>0.955	-0.037<br>0.919	10
**PICP**	-0.177<br>0.737	-0.200<br>0.704	6	0.231<br>0.495	0.136<br>0.689	11	-0.796<br>0.058	-0.928[b]<br>0.008	6	-0.301<br>0.369	-0.200<br>0.555	11
**TGFβ**	-0.658<br>0.155	-0.429<br>0.397	6	-0.287<br>0.491	-0.524<br>0.183	8	0.771<br>0.073	0.841[a]<br>0.036	6	0.428<br>0.290	0.310<br>0.456	8
**VTN**	-0.321<br>0.400	-0.109<br>0.781	9	0.197<br>0.584	-0.006<br>0.987	10	-0.069<br>0.860	-0.059<br>0.881	9	0.536<br>0.110	0.207<br>0.567	10
**C9**	-0.492<br>0.321	-0.486<br>0.329	6	-0.005<br>0.990	-0.115<br>0.751	10	0.226<br>0.667	0.174<br>0.742	6	0.708[a]<br>0.022	0.419<br>0.228	10
**C4BP**			1	0.305<br>0.392	0.309<br>0.385	10			1	0.317<br>0.373	0.103<br>0.776	10
**CRP**	0.445<br>0.128	0.591[a]<br>0.033	13	0.241<br>0.476	0.318<br>0.340	11	-0.217<br>0.477	0.025<br>0.936	13	-0.362<br>0.274	-0.050<br>0.884	11
**FMN**			3	-0.028<br>0.940	-0.080<br>0.827	9			3	0.002<br>0.996	0.084<br>0.831	9
**CFH**			1	0.121<br>0.739	-0.152<br>0.676	10			1	0.355<br>0.314	0.298<br>0.403	10
**CFI**	0.899[a]<br>0.015	0.714<br>0.111	6	-0.028<br>0.940	-0.080<br>0.827	10	-0.043<br>0.935	-0.203<br>0.700	6	-0.127<br>0.726	-0.074<br>0.839	10
**FGG**			0	0.593<br>0.071	0.333<br>0.347	10			0	0.304<br>0.393	0.480<br>0.160	10

Graphical visualization of correlations considered as significant:

Urinary adiponectin (UADP) and serum complement factor I (SCFI) with systolic blood pressure.

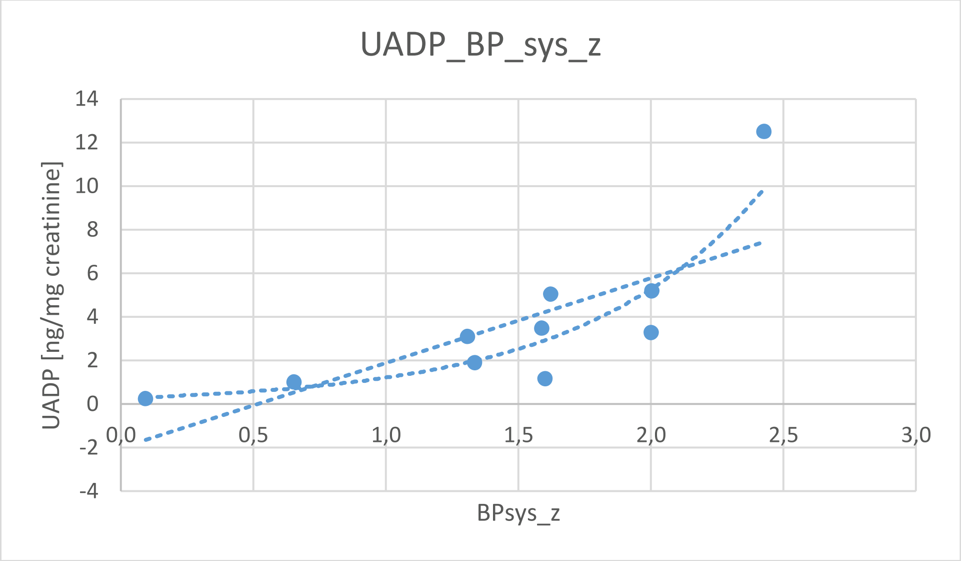

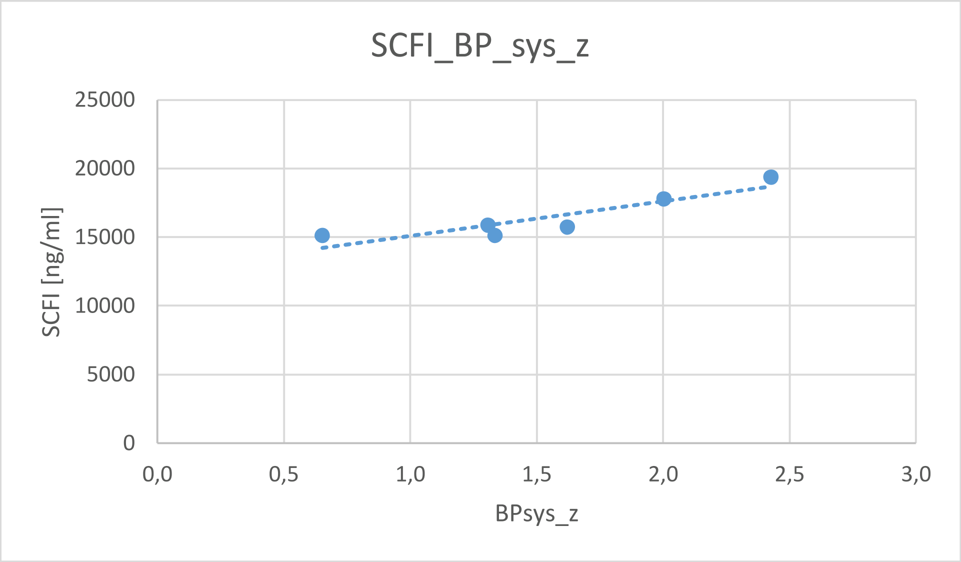

Serum PICP, TGFß, and urinary complement component 9 (UC9) with diastolic blood pressure.

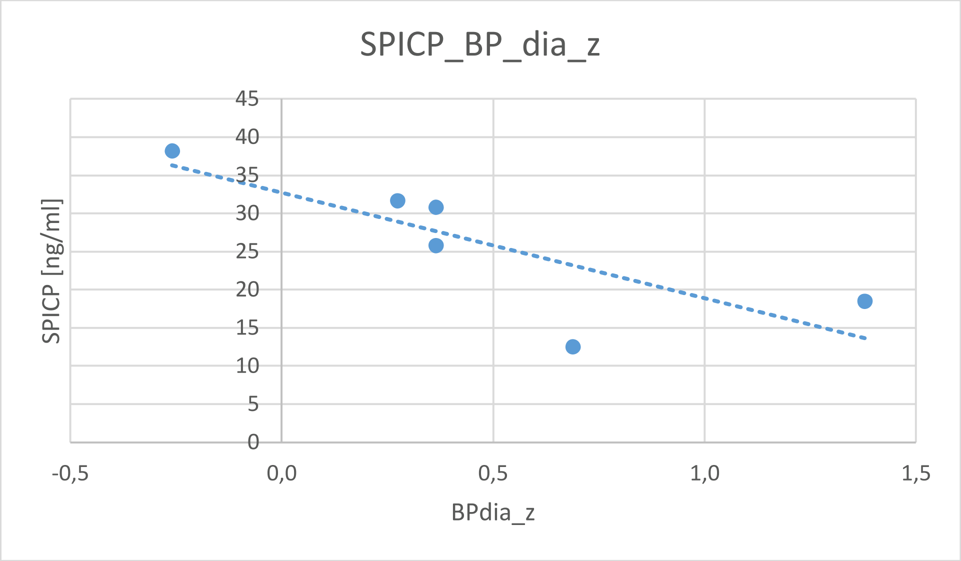

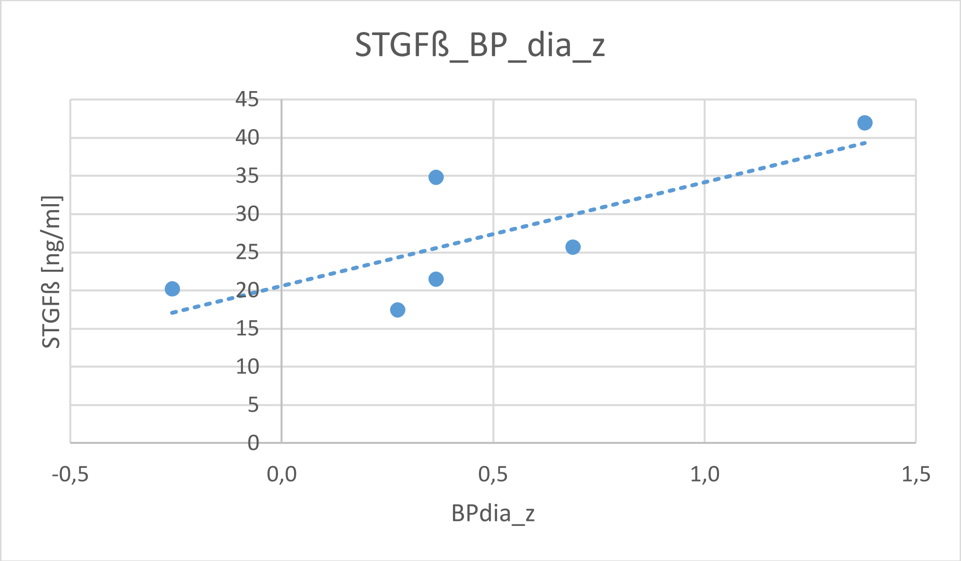

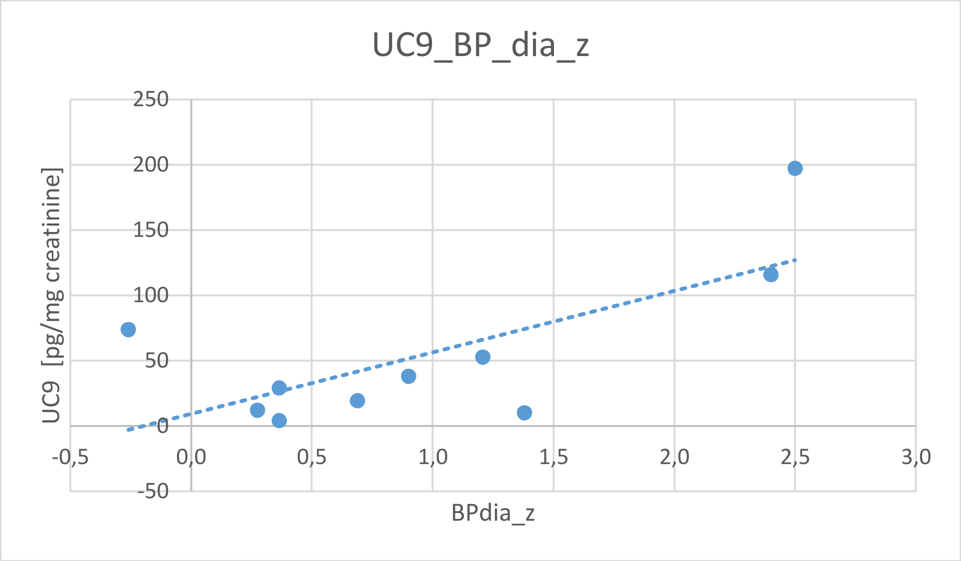

Supplementary Table S16 to S19

Patients with T1DM (group B)

Table S16: Correlation of serum and urinary BM candidates with UACR and blood glucose

	UACR						blood glucose					
**BM in→**	**Serum**			**Urine**			**Serum**			**Urine**		
**↓**	**Pearson**	**Spearman**	**n**	**Pearson**	**Spearman**	**n**	**Pearson**	**Spearman**	**n**	**Pearson**	**Spearman**	**n**
**ADP**			0	0.035<br>0.853	0.064<br>0.733	31			0	0.034<br>0.837	0.167<br>0.304	40
**a1AGP**	0.058<br>0.845	0.096<br>0.744	14	0.947[b]<br><0.001	.488[b]<br>0.005	31	0.733[b]<br>0.002	0.449<br>0.093	15	-0.069<br>0.671	0.091<br>0.578	40
**AGT**			0	-0.169<br>0.362	-0.308<br>0.092	31			0	0.064<br>0.695	-0.134<br>0.411	40
**ColXIII**			0	-0.063<br>0.736	0.083<br>0.655	31			0	-0.149<br>0.365	-0.201<br>0.219	39
**GS**			0	0.086<br>0.650	0.041<br>0.828	30			0	0.041<br>0.803	-0.025<br>0.882	39
**LRGP1**	-0.258<br>0.335	-0.519[a]<br>0.039	16	-0.080<br>0.664	-0.245<br>0.177	32	0.123<br>0.617	0.604[b]<br>0.006	19	0.059<br>0.714	-0.024<br>0.882	41
**HABP2**			0	0.054<br>0.777	0.167<br>0.377	30			0	0.198<br>0.228	0.010<br>0.950	39
**PICP**	-0.032<br>0.940	-0.144<br>0.734	8	0.778[b]<br><0.001	0.495[b]<br>0.005	31	-0.131<br>0.685	0.185<br>0.565	12	0.037<br>0.819	0.250<br>0.119	40
**TGFβ**	-0.138<br>0.745	-0.048<br>0.911	8	-0.254<br>0.183	-0.067<br>0.731	29	0.242<br>0.448	0.105<br>0.745	12	0.057<br>0.738	0.032<br>0.850	37
**VTN**	0.417<br>0.138	0.328<br>0.253	14	-0.099<br>0.590	-0.161<br>0.380	32	0.129<br>0.623	0.247<br>0.339	17	0.011<br>0.946	-0.023<br>0.886	41
**C9**	-0.166<br>0.722	-0.214<br>0.645	7	0.039<br>0.837	0.272<br>0.140	31	-0.362<br>0.304	-0.310<br>0.383	10	-0.038<br>0.818	0.183<br>0.258	40
**C4BP**			0	-0.027<br>0.887	0.129<br>0.489	31			0	-0.067<br>0.682	0.111<br>0.493	40
**CRP**	0.834[b]<br><0.001	0.084<br>0.776	14	-0.019<br>0.920	0.189<br>0.309	31	-0.141<br>0.588	0.006<br>0.981	17	-0.084<br>0.607	0.164<br>0.312	40
**FMN**			0	-0.195<br>0.292	-0.295<br>0.108	31			2	-0.126<br>0.439	-0.148<br>0.363	40
**CFH**			0	-0.031<br>0.870	0.301<br>0.099	31			0	-0.042<br>0.799	0.053<br>0.744	40
**CFI**	-0.040<br>0.926	-0.476<br>0.233	8	-0.026<br>0.888	0.380[a]<br>0.035	31	0.078<br>0.829	0.122<br>0.738	10	0.190<br>0.241	0.140<br>0.389	40
**FGG**			1	-0.104<br>0.577	-0.311<br>0.088	31			3	-0.028<br>0.864	0.006<br>0.971	40

Analyses were performed by SPSS (v.27). Each cell shows the correlation coefficient (upper figure) and a p-value (lower figure).

[a] and [b] indicate significant and highly significant correlations proposed by SPSS. Notwithstanding that, we set a correlation coefficient >0.500 moderately significant and >0.600 highly significant and mark these values by grey filling.

Graphical visualization of correlations considered as significant:

Serum CRP, urinary (U) alpha1-acid glycoprotein (a1AGP), and PICP with UACR, as well as serum (S) a1AGP and leucine rich glycoprotein 1 (LRGP1) with blood glucose

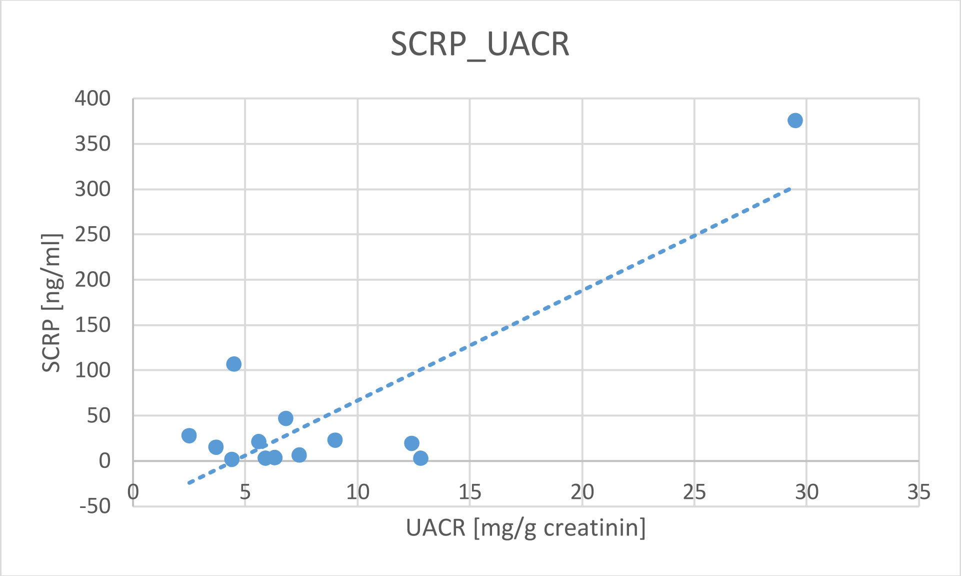

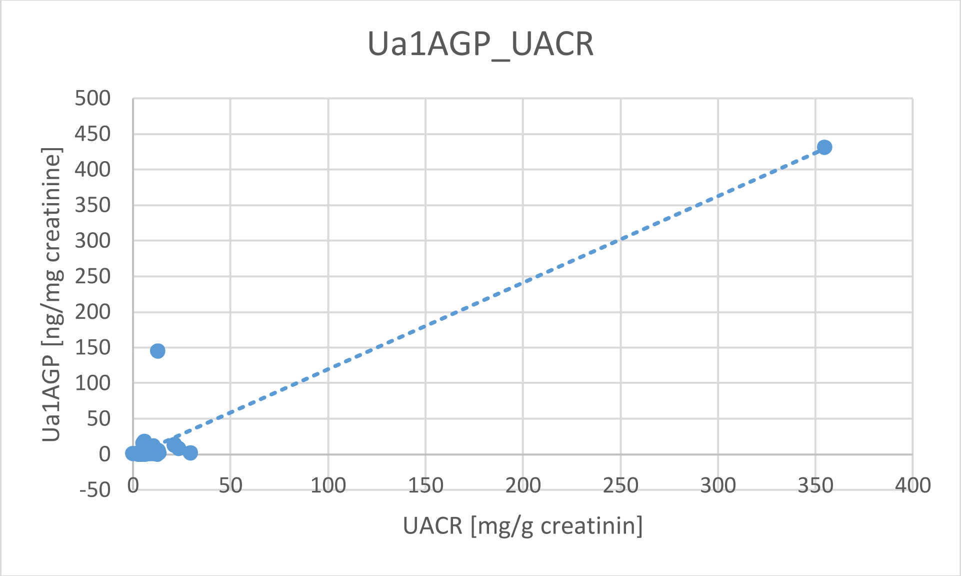

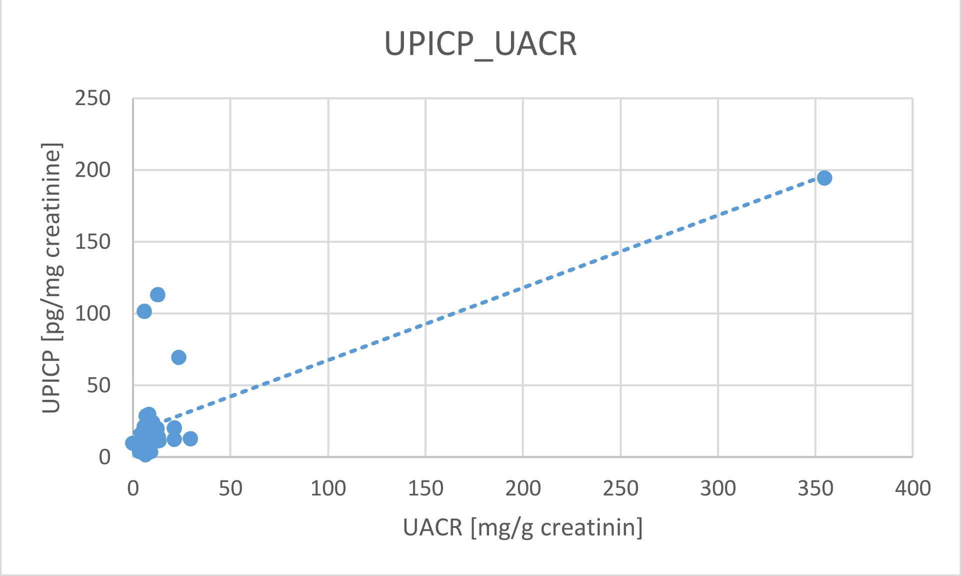

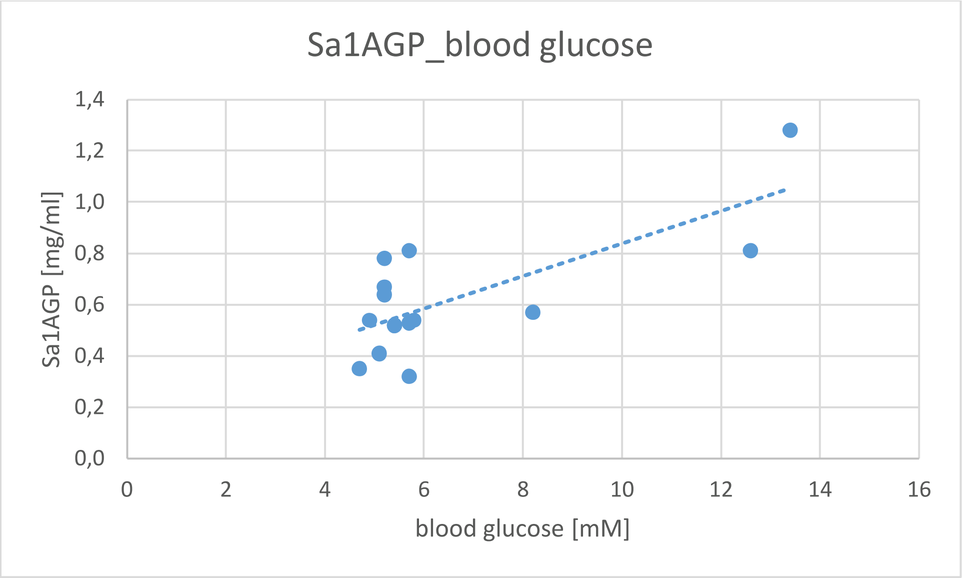

Sa1AGP_blood glucose
Sa1AGP [mg/ml]
1,4
1,2
1,0
0,8
0,6
0,4
0,2
0,0
0
2
4
6
8
10
12
14
16
blood glucose [mM]

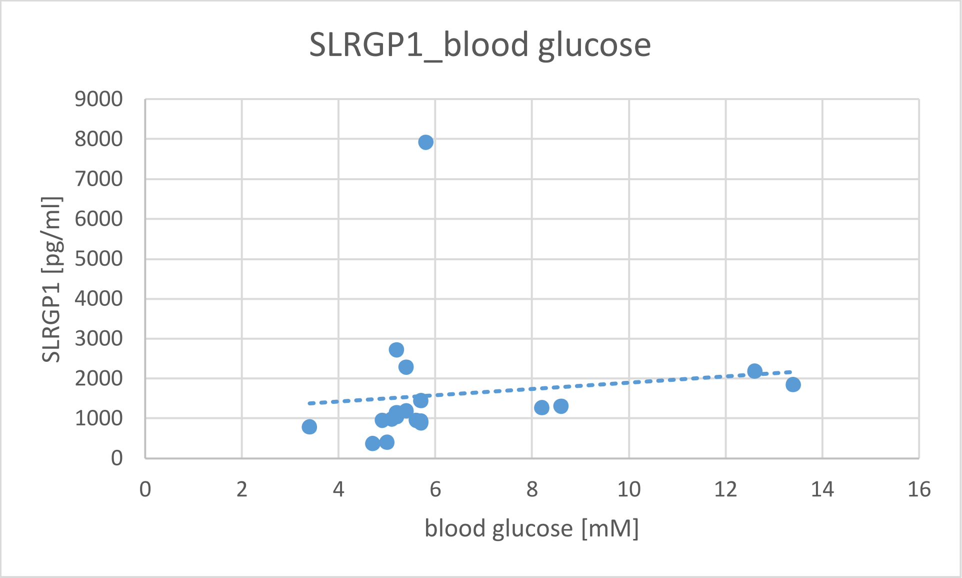

SLRGP1_blood glucose
SLRGP1 [pg/ml]
9000
8000
7000
6000
5000
4000
3000
2000
1000
0
0
2
4
6
8
10
12
14
16
blood glucose [mM]

Table S17: Correlation of serum and urinary BM candidates with HbA1c and BMI z-scores.

	HbA1c						BMI-z-score					
BM in→	Serum			Urine			Serum			Urine		
↓	Pearson	Spearman	n	Pearson	Spearman	n	Pearson	Spearman	n	Pearson	Spearman	n
**ADP**			0	-0.040<br>0.805	0.081<br>0.621	40			0	0.147<br>0.385	0.014<br>0.935	37
**a1AGP**	-0.253<br>0.364	-0.309<br>0.262	15	0.013<br>0.938	-0.040<br>0.808	40	0.004<br>0.987	0.108<br>0.692	16	0.177<br>0.296	0.385[a]<br>0.019	37
**AGT**			0	-0.338[a]<br>0.033	-0.431[b]<br>0.005	40			0	0.190<br>0.259	0.056<br>0.743	37
**ColXIII**			0	-0.056<br>0.734	0.002<br>0.990	39			0	0.260<br>0.126	0.224<br>0.189	36
**GS**			0	-0.359[a]<br>0.025	-0.280<br>0.084	39			0	0.297<br>0.079	0.206<br>0.228	36
**LRGP1**	-0.278<br>0.249	-0.359<br>0.132	19	0.102<br>0.524	-0.015<br>0.928	41	-0.124<br>0.601	0.114<br>0.631	20	0.033<br>0.846	-0.063<br>0.708	38
**HABP2**			0	-0.095<br>0.566	-0.055<br>0.738	39			0	0.096<br>0.576	0.146<br>0.395	36
**PICP**	0.442<br>0.173	0.302<br>0.367	11	-0.110<br>0.500	-0.038<br>0.816	40	0.301<br>0.342	0.053<br>0.871	12	0.320<br>0.053	0.480[b]<br>0.003	37
**TGFβ**	-0.094<br>0.783	-0.192<br>0.572	11	-0.101<br>0.554	-0.230<br>0.172	37	0.132<br>0.683	0.049<br>0.880	12	0.288<br>0.093	0.283<br>0.100	35
**VTN**	-0.188<br>0.470	-0.071<br>0.786	17	-0.431[b]<br>0.005	-0.599[b]<br><.001	41	0.301<br>0.225	0.187<br>0.458	18	0.324[a]<br>0.048	0.282<br>0.087	38
**C9**	-0.241<br>0.502	-0.365<br>0.300	10	-0.268<br>0.095	-0.278<br>0.083	40	0.454<br>0.187	0.200<br>0.580	10	0.276<br>0.098	0.174<br>0.302	37
**C4BP**			0	-0.260<br>0.105	-0.113<br>0.489	40			0	0.229<br>0.173	0.048<br>0.776	37
**CRP**	0.074<br>0.777	0.421<br>0.092	17	-0.112<br>0.492	0.035<br>0.830	40	-0.089<br>0.726	-0.168<br>0.505	18	0.097<br>0.568	0.097<br>0.569	37
**FMN**			2	0.010<br>0.950	-0.147<br>0.367	40			2	-0.079<br>0.640	-0.102<br>0.548	37
**CFH**			0	-0.277<br>0.083	-0.022<br>0.894	40			0	0.248<br>0.139	0.237<br>0.157	37
**CFI**	0.000<br>1.000	-0.195<br>0.590	10	-0.219<br>0.175	-0.195<br>0.229	40	-0.349<br>0.324	-0.442<br>0.200	10	0.308<br>0.064	0.319<br>0.054	37
**FGG**			3	-0.224<br>0.164	-0.281<br>0.079	40			3	0.204<br>0.226	0.141<br>0.405	37

Table S18: Correlation of serum and urinary BM candidates with blood pressure z-scores.

	BP_sys_z-score						BP_dia_z-score					
**BM in→**	**Serum**			**Urine**			**Serum**			**Urine**		
**↓**	**Pearson**	**Spearman**	**n**	**Pearson**	**Spearman**	**n**	**Pearson**	**Spearman**	**n**	**Pearson**	**Spearman**	**n**
**ADP**			0	-0.387[a]<br>0.022	-0.423[a]<br>0.011	35			0	-0.189<br>0.278	-0.204<br>0.239	35
**a1AGP**	-0.257<br>0.375	-0.137<br>0.642	14	-0.069<br>0.694	0.074<br>0.673	35	0.005<br>0.986	0.066<br>0.822	14	0.009<br>0.958	0.133<br>0.445	35
**AGT**			0	0.071<br>0.684	0.096<br>0.585	35			0	-0.126<br>0.470	-0.086<br>0.624	35
**ColXIII**			0	0.203<br>0.249	0.083<br>0.643	34			0	-0.073<br>0.681	-0.043<br>0.810	34
**GS**			0	0.057<br>0.747	0.208<br>0.238	34			0	-0.109<br>0.540	-0.062<br>0.729	34
**LRGP1**	-0.008<br>0.973	0.356<br>0.147	18	0.209<br>0.221	-0.260<br>0.126	36	-0.417<br>0.085	0.124<br>0.624	18	0.184<br>0.282	-0.065<br>0.706	36
**HABP2**			0	-0.301<br>0.083	-0.338<br>0.051	34			0	-0.032<br>0.859	-0.040<br>0.824	34
**PICP**	-0.253<br>0.453	-0.068<br>0.842	11	-0.056<br>0.749	0.016<br>0.929	35	-0.282<br>0.401	-0.048<br>0.889	11	0.071<br>0.687	0.380[a]<br>0.024	35
**TGFβ**	0.102<br>0.765	0.064<br>0.853	11	-0.044<br>0.810	0.016<br>0.928	33	0.196<br>0.563	0.114<br>0.739	11	-0.249<br>0.163	-0.246<br>0.168	33
**VTN**	0.196<br>0.468	0.100<br>0.713	16	0.137<br>0.427	0.216<br>0.205	36	0.285<br>0.285	0.272<br>0.308	16	-0.016<br>0.924	0.044<br>0.800	36
**C9**	0.646<br>0.060	0.467<br>0.205	9	0.054<br>0.757	0.042<br>0.809	35			9	0.095<br>0.586	0.082<br>0.641	35
**C4BP**			0	0.070<br>0.690	0.173<br>0.322	35			0	0.061<br>0.728	0.191<br>0.272	35
**CRP**	0.043<br>0.876	0.256<br>0.339	16	-0.057<br>0.747	0.050<br>0.773	35	-0.025<br>0.928	0.222<br>0.408	16	-0.007<br>0.969	0.142<br>0.417	35
**FMN**			2	0.315<br>0.065	0.210<br>0.225	35			2	0.021<br>0.904	-0.033<br>0.850	35
**CFH**			0	-0.059<br>0.734	0.052<br>0.767	35			0	-0.090<br>0.608	-0.034<br>0.848	35
**CFI**	-0.200<br>0.606	-0.117<br>0.765	9	-0.040<br>0.821	0.125<br>0.476	35	-0.256<br>0.505	-0.151<br>0.699	9	-0.022<br>0.902	0.096<br>0.585	35
**FGG**			2	0.246<br>0.155	0.284<br>0.099	35			2	-0.051<br>0.770	-0.035<br>0.843	35

Table S19: Correlation of serum and urinary BM candidates with disease duration.

	Disease duration [years]					
BM in→	Serum			Urine		
↓	Pearson	Spearman	n	Pearson	Spearman	n
**ADP**			0	-0.080<br>0.616	-0.161<br>0.308	42
**a1AGP**	-0.361<br>0.170	-0.178<br>0.509	16	-0.018<br>0.908	0.078<br>0.625	42
**AGT**			0	-0.073<br>0.646	0.011<br>0.943	42
**ColXIII**			0	0.005<br>0.976	0.046<br>0.776	41
**GS**			0	-0.088<br>0.585	-0.123<br>0.443	41
**LRGP1**	-0.166<br>0.485	-0.059<br>0.806	20	-0.143<br>0.361	-0.142<br>0.362	43
**HABP2**			0	0.172<br>0.282	0.088<br>0.586	41
**PICP**	-0.293<br>0.355	-0.364<br>0.245	12	0.006<br>0.971	-0.031<br>0.845	43
**TGFβ**	-0.242<br>0.449	-0.207<br>0.519	12	-0.292<br>0.071	-0.279<br>0.085	39
**VTN**	0.492[a]<br>0.038	0.428<br>0.076	18	-0.025<br>0.872	0.001<br>0.997	43
**C9**	-0.226<br>0.504	-0.145<br>0.670	11	-0.060<br>0.707	-0.126<br>0.428	42
**C4BP**			0	-0.019<br>0.907	0.004<br>0.982	42
**CRP**	0.593[b]<br>0.010	0.471[a]<br>0.049	18	-0.033<br>0.834	0.257<br>0.093	44
**FMN**			2	-0.233<br>0.138	-0.181<br>0.251	42
**CFH**			0	-0.025<br>0.875	-0.176<br>0.264	42
**CFI**	-0.338<br>0.309	-0.487<br>0.128	11	-0.033<br>0.835	0.045<br>0.777	42
**FGG**			3	-0.054<br>0.732	-0.045<br>0.778	42

Supplementary Tables S20 to S23

Patients with nephropathies (groups A to G)

Table S20: Correlation of serum and urinary BM candidates with serum creatinine and UACR

	Serum creatinine						UACR					
**BM in→**	**Serum**			**Urine**			**Serum**			**Urine**		
**↓**	**Pearson**	**Spearman**	**n**	**Pearson**	**Spearman**	**n**	**Pearson**	**Spearman**	**n**	**Pearson**	**Spearman**	**n**
**ADP**	0.041	0.015	34	-0.065	-0.134	80	0.147	0.407[b]	66	0.092	0.012	114
	0.819	0.932		0.567	0.237		0.240	<0.001		0.332	0.897	
**a1AGP**	-0.129	0.004	29	0.058	0.029	75	-0.032	0.214	49	0.199[a]	0.583[b]	109
	0.505	0.983		0.621	0.807		0.830	0.141		0.038	<0.001	
**AGT**	0.183	0.185	33	0.021	0.077	73	0.133	-0.158	65	0.115	0.342[b]	107
	0.308	0.302		0.859	0.519		0.290	0.209		0.238	<0.001	
**ColXIII**	0.040	0.038	33	0.115	0.074	74	-0.025	0.150	65	0.279[b]	0.353[b]	108
	0.827	0.832		0.329	0.531		0.845	0.232		0.003	<0.001	
**GS**	-0.042	0.026	34	0.040	0.067	63	0.151	0.290[a]	66	0.128	0.382[b]	96
	0.815	0.884		0.756	0.604		0.225	0.018		0.214	<0.001	
**LRGP1**	0.136	0.060	66	-0.115	-0.108	80	-0.058	0.166	100	0.271[b]	0.571[b]	114
	0.275	0.631		0.310	0.338		0.565	0.100		0.004	<0.001	
**HABP2**	-0.126	-0.132	35	0.073	0.019	63	0.260[a]	0.261[a]	67	0.231[a]	0.399[b]	96
	0.470	0.450		0.570	0.881		0.034	0.033		0.023	<0.001	
**PICP**	-0.097	-0.060	51	0.001	0.066	86	-0.019	0.419[b]	85	0.113	0.379[b]	116
	0.499	0.674		0.993	0.547		0.866	<0.001		0.227	<0.001	
**TGFβ**	-0.231	-0.217	50	-0.116	-0.026	67	-0.039	0.281[a]	81	0.023	0.171	102
	0.107	0.131		0.349	0.832		0.730	0.011		0.818	0.086	
**VTN**	0.108	0.102	61	-0.115	-0.055	77	-0.219[a]	-0.335[b]	94	0.052	0.255[b]	111
	0.405	0.436		0.318	0.637		0.034	<0.001		0.586	0.007	
**C9**	-0.251	-0.118	17	0.014	-0.045	72	-0.006	0.198	53	0.202[a]	0.491[b]	106
	0.330	0.653		0.909	0.710		0.968	0.160		0.038	<0.001	
**C4BP**	-0.253	-0.112	33	0.035	0.074	76	-0.036	0.003	65	0.359[b]	0.635[b]	110
	0.155	0.534		0.764	0.523		0.776	0.982		<0.001	<0.001	
**CRP**	0.003	-0.152	82	-0.007	-0.065	86	-0.040	0.115	116	0.014	0.404[b]	117
	0.977	0.172		0.946	0.550		0.668	0.218		0.878	<0.001	
**LUM**			1			1	0.188	0.090	22	0.680[b]	0.580[b]	22
							0.402	0.691		<0.001	0.005	
**FMN**	0.015	0.001	36	-0.003	-0.012	78	-0.068	0.058	66	0.242[a]	0.425[b]	107
	0.930	0.995		0.982	0.914		0.589	0.641		0.012	<0.001	
**CFH**	-0.092	-0.371	6	0.028	0.052	69	0.056	0.498[a]	25	0.256[b]	0.714[b]	103
	0.863	0.468		0.817	0.671		0.792	0.011		0.009	<0.001	
**CFI**	-0.308	-0.261	17	0.116	0.112	71	0.330[a]	0.464[b]	38	0.208[a]	0.588[b]	105
	0.229	0.311		0.335	0.351		0.043	0.003		0.033	<0.001	
**FGG**	0.146	0.155	35	-0.099	-0.089	79	-0.093	-0.106	35	0.287[b]	0.475[b]	109
	0.403	0.374		0.385	0.436		0.596	0.545		0.002	<0.001	
**C1q**	-0.217	-0.162	36	-0.041	0.072	37	0.008	-0.097	68	-0.015	0.612[b]	65
	0.203	0.344		0.810	0.671		0.950	0.432		0.907	<0.001	

Analyses were performed by SPSS (v.27). Each cell shows the correlation coefficient (upper figure) and a P-value (lower figure).

[a] and [b] indicate significant and highly significant correlations proposed by SPSS. Notwithstanding that, we set a correlation coefficient >0.500 moderately significant and >0.600 highly significant and mark these values by grey filling.

Graphical visualization of correlation considered as significant:

Urinary lumican (ULUM) with UACR

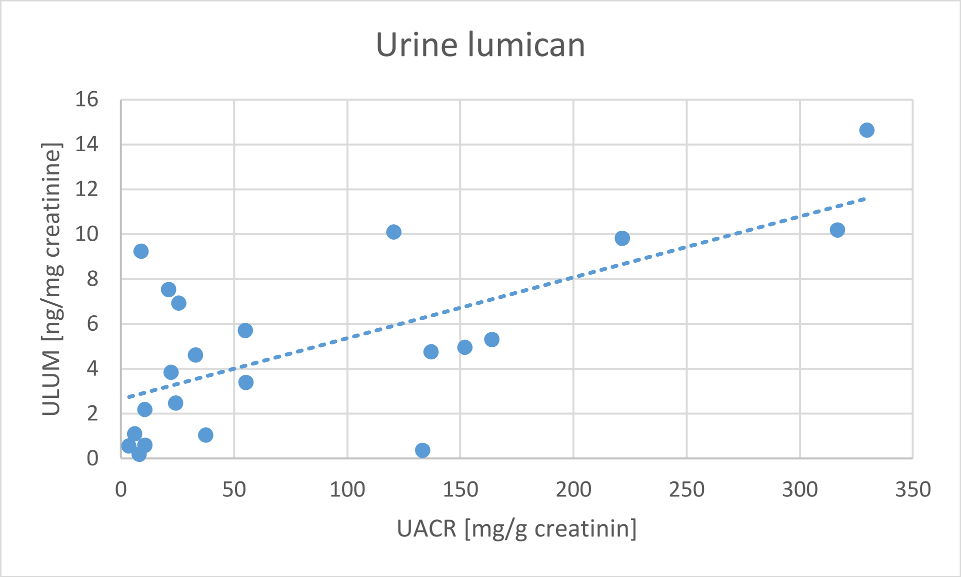

Table S21: Correlation of serum and urinary BM candidates with eGFR and cGFR.

	eGFR						cGFR					
BM in→	Serum			Urine			Serum			Urine		
↓	Pearson	Spearman	n	Pearson	Spearman	n	Pearson	Spearman	n	Pearson	Spearman	n
ADP	-0.014<br>0.939	-0.080<br>0.654	34	0.097<br>0.404	0.205<br>0.075	76	-0.136<br>0.457	-0.017<br>0.927	32	-0.034<br>0.768	0.038<br>0.746	76
a1AGP	0.553[b]<br>0.004	0.371<br>0.068	25	-0.026<br>0.831	0.063<br>0.602	71	0.050<br>0.797	-0.139<br>0.471	29	-0.121<br>0.311	-0.062<br>0.602	72
AGT	-0.310<br>0.079	-0.230<br>0.198	33	-0.032<br>0.792	-0.030<br>0.805	69	0.339<br>0.062	0.111<br>0.553	31	-0.062<br>0.608	0.014<br>0.908	70
ColXIII	-0.207<br>0.248	-0.153<br>0.395	33	-0.077<br>0.525	0.010<br>0.937	70	-0.233<br>0.207	-0.275<br>0.134	31	-0.055<br>0.651	0.028<br>0.818	71
GS	0.086<br>0.631	0.015<br>0.932	34	0.025<br>0.852	0.098<br>0.460	59	-0.061<br>0.739	-0.108<br>0.557	32	-0.036<br>0.786	0.087<br>0.509	60
LRGP1	-0.038<br>0.768	-0.047<br>0.714	62	0.059<br>0.615	0.130<br>0.264	76	-0.218<br>0.086	-0.202<br>0.112	63	-0.020<br>0.861	-0.027<br>0.815	76
HABP2	0.149<br>0.393	0.109<br>0.532	35	-0.028<br>0.831	0.067<br>0.612	59	-0.330<br>0.061	-0.286<br>0.107	33	0.013<br>0.923	0.128<br>0.330	60
PICP	0.126<br>0.388	0.221<br>0.128	49	-0.009<br>0.939	-0.026<br>0.816	82	-0.141<br>0.334	-0.132<br>0.367	49	-0.009<br>0.935	0.001<br>0.994	82
TGFβ	0.218<br>0.137	0.104<br>0.480	48	0.032<br>0.804	-0.026<br>0.840	63	-0.074<br>0.617	-0.125<br>0.398	48	0.041<br>0.751	0.075<br>0.557	63
VTN	-0.084<br>0.536	-0.072<br>0.596	57	0.096<br>0.421	-0.015<br>0.897	73	0.207<br>0.120	0.080<br>0.548	58	0.038<br>0.749	-0.064<br>0.590	74
C9	-0.379<br>0.148	-0.415<br>0.110	16	-0.020<br>0.871	0.036<br>0.770	69	0.116<br>0.657	0.104<br>0.690	17	-0.062<br>0.613	0.059<br>0.631	69
C4BP	0.165<br>0.359	0.150<br>0.403	33	-0.001<br>0.992	-0.026<br>0.831	72	-0.184<br>0.323	-0.380[a]<br>0.035	31	-0.039<br>0.741	-0.163<br>0.167	73
CRP	0.009<br>0.936	0.127<br>0.268	78	0.087<br>0.439	0.149<br>0.182	82	-0.060<br>0.601	-0.147<br>0.199	78	-0.033<br>0.771	-0.007<br>0.953	82
LUM			1			1			1			1
FMN	0.085<br>0.622	0.071<br>0.680	36	0.030<br>0.799	-0.017<br>0.889	74	-0.095<br>0.595	-0.096<br>0.588	34	-0.066<br>0.574	-0.076<br>0.518	74
CFH	0.240<br>0.647	0.829[a]<br>0.042	6	-0.020<br>0.873	-0.025<br>0.843	65	-0.735<br>0.096	-0.771<br>0.072	6	-0.019<br>0.877	-0.065<br>0.605	66
CFI	0.260<br>0.330	-0.003<br>0.991	16	-0.048<br>0.701	-0.018<br>0.887	67	-0.369<br>0.145	-0.311<br>0.224	17	-0.079<br>0.523	-0.013<br>0.914	68
FGG	-0.067<br>0.702	-0.067<br>0.703	35	0.128<br>0.274	0.146<br>0.211	75	0.025<br>0.891	-0.075<br>0.677	33	0.078<br>0.505	-0.017<br>0.883	76
C1q	0.227<br>0.184	0.317<br>0.060	36	0.002<br>0.990	-0.033<br>0.844	37	-0.234<br>0.182	-0.260<br>0.137	34	-0.014<br>0.937	0.168<br>0.336	35

Graphical visualization of correlations considered as significant:

Serum complement factor H with eGFR and cGFR

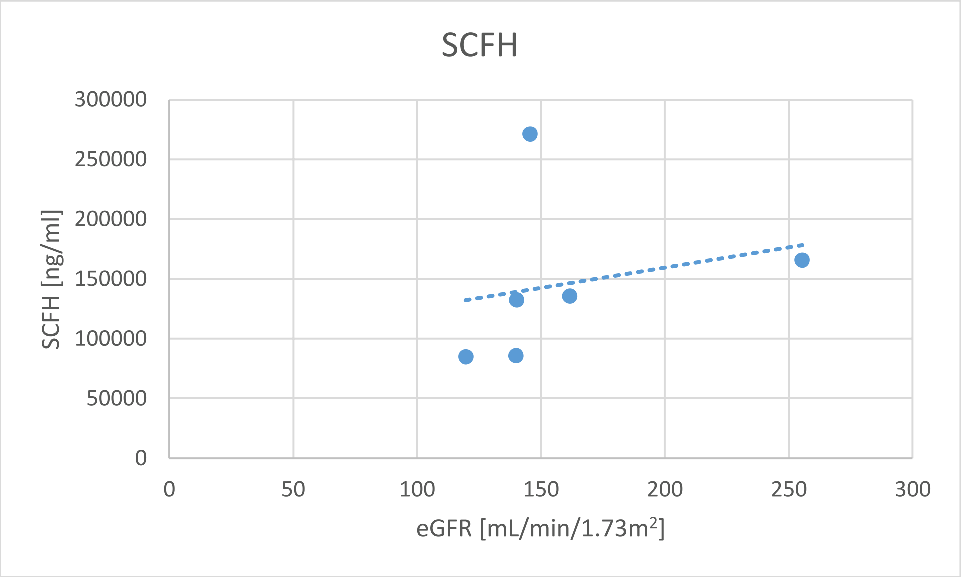

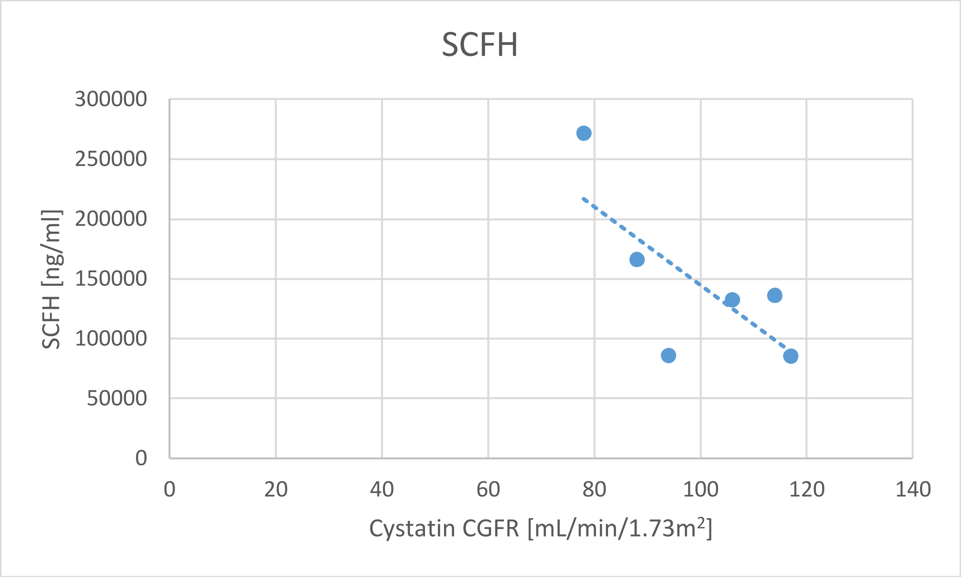

Table S22: Correlation of serum and urinary BM candidates with serum CRP

	SCRP					
BM in→	Serum			Urine		
↓	Pearson	Spearman	n	Pearson	Spearman	n
**ADP**	-0.082	-0.120	70	-0.086	-0.194[a]	116
	0.501	0.322		0.359	0.037	
**a1AGP**	0.279[a]	0.398[b]	52	0.031	0.424[b]	111
	0.045	0.003		0.746	<0.001	
**AGT**	-0.153	-0.086	69	-0.026	0.176	109
	0.209	0.482		0.785	0.068	
**ColXIII**	-0.039	0.233	69	0.380[b]	0.493[b]	110
	0.753	0.054		<0.001	<0.001	
**GS**	-0.071	0.146	70	0.007	0.216[a]	98
	0.557	0.229		0.946	0.033	
**LRGP1**	0.072	0.298[b]	106	0.360[b]	0.561[b]	116
	0.463	0.002		<0.001	<0.001	
**HABP2**	0.276[a]	0.517[b]	71	0.027	0.419[b]	98
	0.020	<0.001		0.792	<0.001	
**PICP**	0.199	0.220[a]	90	-0.095	-0.110	121
	0.060	0.037		0.301	0.229	
**TGFβ**	0.138	0.479[b]	86	-0.025	-0.026	103
	0.206	<0.001		0.804	0.795	
**VTN**	-0.155	-0.116	100	-0.061	-0.005	113
	0.123	0.250		0.522	0.955	
**C9**	-0.067	0.286[a]	54	-0.035	0.194[a]	108
	0.631	0.036		0.717	0.045	
**C4BP**	0.140	0.328[b]	69	0.087	0.573[b]	112
	0.250	0.006		0.364	<0.001	
**SCRP**	1.000	1.000	122	0.532[b]	0.612[b]	177
				<0.001	<0.001	
**LUM**	0.365	0.311	23	0.009	0.557[b]	23
	0.087	0.148		0.968	0.006	
**FMN**	-0.117	-0.070	70	0.124	0.499[b]	112
	0.336	0.567		0.191	<0.001	
**CFH**	0.634[b]	0.493[a]	26	0.233[a]	0.600[b]	105
	<0.001	0.010		0.017	<0.001	
**CFI**	0.065	0.375[a]	39	0.025	0.351[b]	107
	0.696	0.019		0.802	<0.001	
**FGG**	-0.453[b]	-0.152	37	0.223[a]	0.514[b]	112
	0.005	0.368		0.018	<0.001	
**C1q**	0.217	0.412[b]	72	0.509[b]	0.621[b]	68
	0.068	<0.001		<0.001	<0.001	

Graphical visualization of correlation considered as significant:

Serum complement factor H with serum CRP

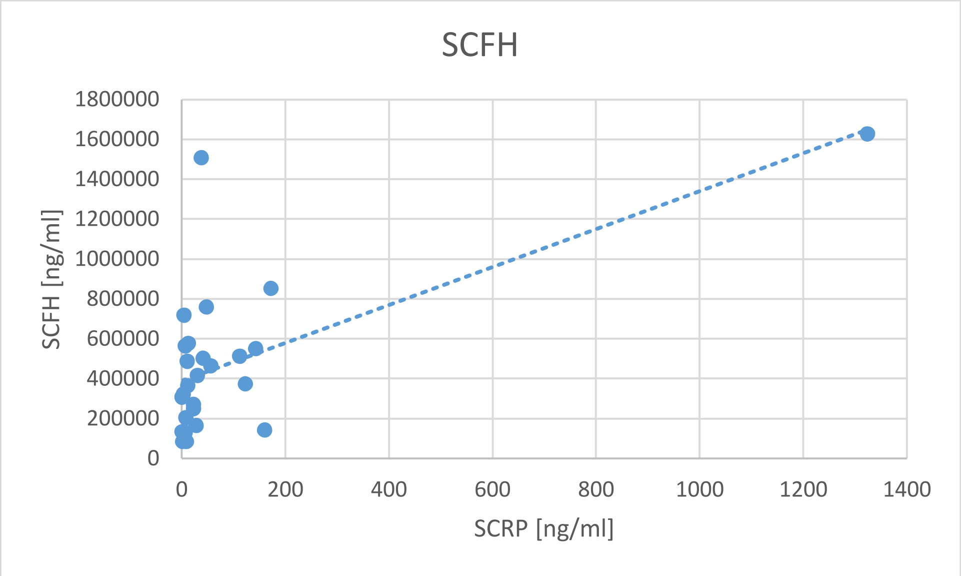

Table S23: Correlation of serum and urinary BM candidates with blood pressure (BP z-score)

	BP_sys_z-score						BP_dia_z-score					
BM in→	Serum			Urine			Serum			Urine		
↓	Pearson	Spearman	n	Pearson	Spearman	n	Pearson	Spearman	n	Pearson	Spearman	n
**ADP**	-0.054	-0.048	30	-0.204	-0.218	71	0.001	-0.007	30	-0.227	-0.163	71
	0.777	0.801		0.087	0.068		0.995	0.971		0.057	0.175	
**a1AGP**	-0.133	-0.167	25	-0.065	-0.156	68	-0.236	-0.235	25	0.074	-0.150	68
	0.525	0.424		0.599	0.205		0.256	0.258		0.546	0.222	
**AGT**	0.121	0.081	29	-0.026	0.028	66	0.187	0.218	29	-0.146	-0.072	66
	0.532	0.677		0.837	0.822		0.330	0.255		0.243	0.568	
**ColXIII**	0.120	0.136	29	0.128	-0.040	67	0.341	0.259	29	0.011	-0.019	67
	0.535	0.481		0.303	0.745		0.070	0.175		0.927	0.876	
**GS**	-0.024	0.014	30	-0.017	0.047	55	0.118	0.107	30	-0.159	-0.043	55
	0.899	0.943		0.902	0.733		0.535	0.575		0.247	0.758	
**LRGP1**	-0.119	0.005	59	-0.089	-0.209	71	-0.175	0.010	59	-0.038	-0.217	71
	0.371	0.969		0.462	0.080		0.186	0.938		0.752	0.069	
**HABP2**	-0.045	-0.085	31	-0.029	-0.034	55	0.027	0.060	31	-0.164	-0.145	55
	0.809	0.650		0.833	0.807		0.886	0.750		0.232	0.291	
**PICP**	-0.114	0.085	45	0.145	0.110	76	0.069	0.034	45	0.107	0.150	76
	0.458	0.577		0.210	0.346		0.653	0.822		0.356	0.195	
**TGFβ**	-0.217	-0.176	43	0.212	0.277	58	-0.089	-0.114	43	0.344	0.384	58
	0.163	0.259		0.110	0.035		0.568	0.466		0.008	0.003	
**VTN**	0.040	0.014	53	-0.117	-0.060	70	0.048	-0.007	53	0.023	-0.010	70
	0.777	0.921		0.335	0.623		0.732	0.960		0.849	0.932	
**C9**	-0.014	0.011	14	-0.163	-0.081	65	-0.111	-0.198	14	-0.132	-0.038	65
	0.961	0.970		0.195	0.523		0.705	0.497		0.296	0.761	
**C4BP**	-0.113	-0.237	29	-0.116	-0.123	69	-0.048	-0.128	29	-0.191	-0.117	69
	0.558	0.216		0.343	0.313		0.803	0.509		0.115	0.340	
**CRP**	0.087	-0.012	73	0.056	-0.118	76	-0.019	-0.187	73	-0.146	-0.353	76
	0.466	0.922		0.632	0.312		0.876	0.114		0.208	0.002	
**FMN**	0.095	0.119	32	-0.004	-0.162	69	-0.388	-0.160	32	-0.109	-0.149	69
	0.605	0.516		0.973	0.184		0.028	0.382		0.373	0.221	
**CFH**	0.179	0.300	5	-0.065	-0.019	62	-0.320	0.100	5	-0.165	0.092	62
	0.773	0.624		0.617	0.886		0.599	0.873		0.201	0.477	
**CFI**	-0.216	-0.332	14	0.010	0.052	64	-0.266	-0.387	14	-0.059	-0.004	64
	0.459	0.246		0.940	0.684		0.359	0.171		0.646	0.976	
**FGG**	0.216	0.251	31	0.026	-0.004	71	0.063	0.124	31	-0.031	-0.059	71
	0.244	0.173		0.828	0.975		0.738	0.507		0.799	0.627	
**C1q**	0.101	0.069	32	0.018	0.237	33	-0.057	-0.068	32	-0.291	-0.007	33
	0.581	0.706		0.921	0.183		0.755	0.711		0.100	0.967	

Supplementary Tables S24 and S25: Analysis of sex differences of BM

Table S24: Significance (P-values) of sex differences of urinary BM in various groups

**BM**	**control group**		**groups C to G**		**group A**		**group B**	
	P-value	n (m/f)	P-value	n (m/f)	P-value	n (m/f)	P-value	n (m/f)
ADP	0.923	45/56	0.681	40/33	0.435	5/8	0.819	21/22
**a1AGP**	**0.021**	44/56	0.371	38/32	0.943	5/8	0.914	21/22
AGT	0.94	41/55	0.743	37/31	0.524	5/8	0.275	21/22
ColXIII	0.934	45/55	0.591	38/31	0.567	4/6	0.905	20/22
GS	0.915	43/56	0.742	29/28	0.284	5/8	0.22	21/21
LRGP1	0.522	45/56	0.582	40/33	0.752	5/8	0.52	21/22
HABP2	0.857	42/56	0.640	29/28	0.131	5/8	0.171	22/22
PICP	0.813	45/56	0.773	44/34	0.298	5/9	0.354	21/21
TGFß	0.486	44/49	0.400	34/26	0.398	5/6	0.941	20/20
VTN	0.443	45/56	0.998	39/33	0.724	5/8	0.121	22/22
C9	0.834	43/56	0.227	36/31	0.943	5/8	0.801	21/22
**C4BP**	**0.010**	44/56	0.841	38/33	0.524	5/8	**0.018**	21/22
CRP	0.889	45/56	0.367	44/34	0.364	5/9	0.719	23/22
CFH	0.036	43/56	0.833	35/29	0.435	5/8	0.334	21/22
CFI	0.547	43/56	0.633	30/36	0.801	5/8	0.832	21/22
FMN	0.996	45/55	0.911	38/33	0.808	4/8	0.195	21/22
**FGG**	0.826	45/56	0.118	40/33	0.622	5/8	**0.011**	21/22
C1q	0.270	40/46	0.414	16/17				

Table S25: Significance (p-values) of sex differences of serum BM in various groups

**BM**	**control group**		**groups C to G**		**group A**		**group B**	
	P-value	n (m/f)	P-value	n (m/f)	P-value	n (m/f)	P-value	n (m/f)
ADP	0.727	3/9	0.984	16/15				
a1AGP	0.902	6/11	0.848	15/11	0.135	5/5	0.905	7/10
AGT	0.482	3/9	0.240	16/14				
ColXIII	0.727	3/9	0.157	16/14				
GS	1.00	3/9	0.067	16/15				
LRGP1	0.978	9/16	0.685	33/28	0.492	6/10	0.918	10/11
HABP2	0.864	3/9	0.455	17/15				
PICP	0.539	5/15	0.849	22/24	0.629	3/ 4	0.034	6/7
TGFß	0.497	5/15	0.788	20/24	0.400	3/4	0.445	6/7
VTN	0.925	8/15	0.205	28/27	0.792	5/6	0.243	10/9
C9	0.222	1/8	0.181	6/8	0.400	3/4	0.247	5/6
C4BP	0.282	3/9	0.667	16/14				
CRP	0.053	10/16	0.145	41/34	0.961	6/11	0.842	9/10
CFH	0.667	1/ 2	0.005	3/2				
CFI	0.667	2/7	0.800	7/7	0.400	3/4	0.537	5/6
FMN	1.00	4/9	0.620	16/17	0.667	1/2		
FGG	0.240	5/9	0.766	40/33				
C1q	0.630	3/8	0.576	15/17				

Analyses were performed by SPSS (v.27). m, males; f, females.
BMs that appear significantly different between males and females are indicated by bold letters and are shown graphically (see below). Empty cells: not enough values for analysis.

Control group: urinary C4BP

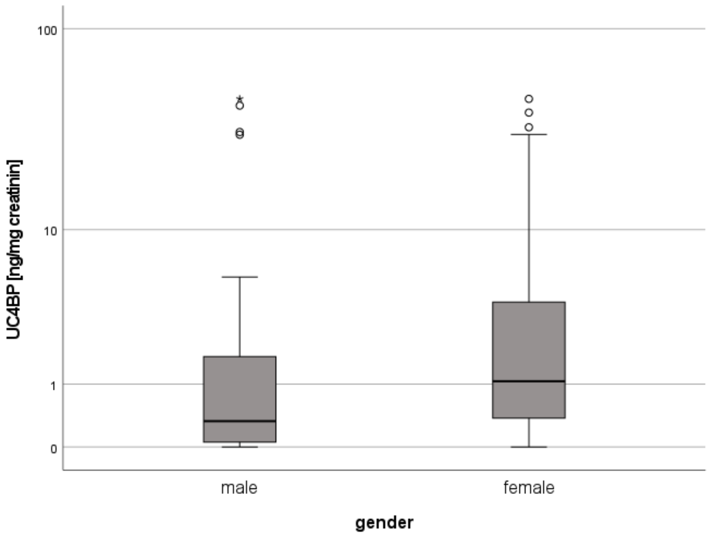

Control group: urinary a1AGP

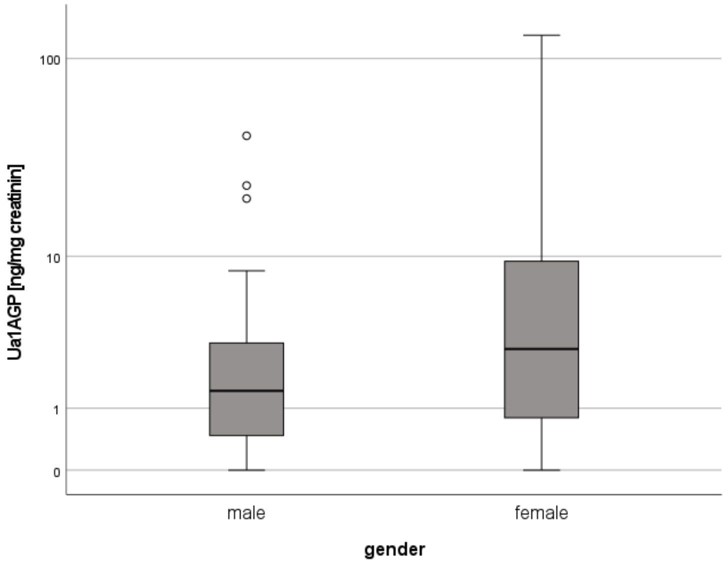

T1DM, group B: urinary C4BP and FGG

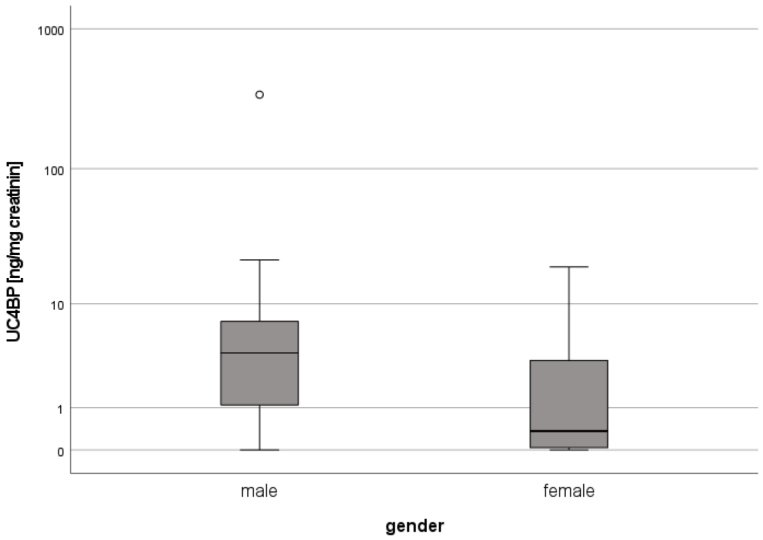

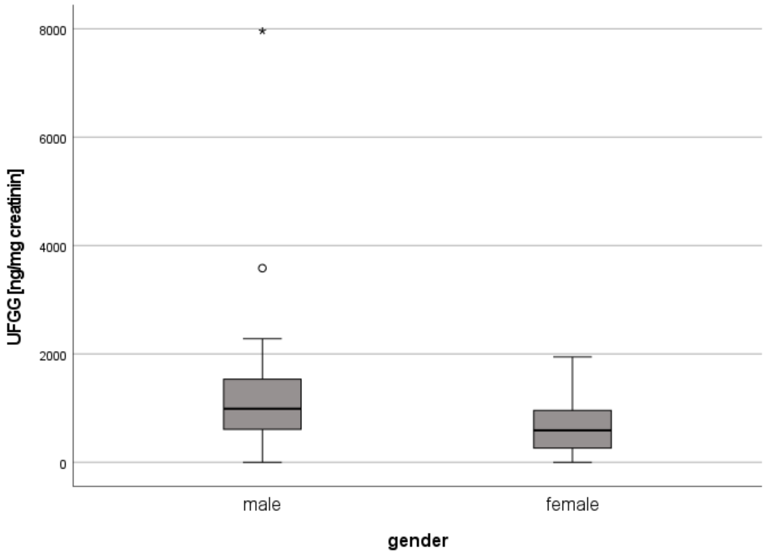

Supplement: Supplementary file 1 [file ijms-25-09320-s001.zip › ijms-3139696-supplementary.pdf]
